# Pan-Cancer Integrated Analysis Identification of SASH3, a Potential Biomarker That Inhibits Lung Adenocarcinoma Progression

**DOI:** 10.3389/fonc.2022.927988

**Published:** 2022-06-03

**Authors:** Xi Chen, Yixiao Yuan, Wenjun Ren, Fan Zhou, Xiaobin Huang, Jun Pu, Xiaoqun Niu, Xiulin Jiang

**Affiliations:** ^1^ Department of Neurosurgery, The Second Affiliated Hospital of Kunming Medical University, Kunming, China; ^2^ Department of Thoracic Surgery, The Third Affiliated Hospital of Kunming Medical University, Kunming, China; ^3^ Department of Respiratory Medicine, The 2nd Affiliated Hospital of Kunming Medical University, Kunming, China; ^4^ Department of Cardiovascular Surgery, The First People’s Hospital of Yunnan Province, Kunming, China; ^5^ Hematology and Rheumatology Department, The Pu’er People’s Hospital, Pu’er, China; ^6^ Key Laboratory of Animal Models and Human Disease Mechanisms of Chinese Academy of Sciences and Yunnan Province, Kunming Institute of Zoology, Kunming, China; ^7^ Kunming College of Life Science, University of Chinese Academy of Sciences, Beijing, China

**Keywords:** SASH3, pan-cancer, immune cell infiltration, prognosis biomarker, LUAD, malignant phenotypes

## Abstract

Sterile alpha motif (SAM) and Src homology-3 (SH3) domain-containing 3 (SASH3) is an adaptor protein expressed mainly in lymphocytes, and plays significant roles in T-cell proliferation and cell survival. However, its expression level, clinical significance, and correlation with tumor-infiltrating immune cells across cancers remain unclear. In this study, we comprehensively examined the expression, dysregulation, and prognostic significance of SASH3, and the correlation with clinicopathological parameters and immune infiltration in pan-cancer. The mRNA and protein expression status of SASH3 were determined by TCGA, GTEx, and UALCAN. Kaplan–Meier analysis utilized the prognostic values of SASH3 in diverse cancers. The association between SASH3 expression and gene mutation, DNA methylation, immune cells infiltration, immune checkpoints, tumor mutation burden (TMB), and microsatellite instability (MSI) were analyzed using data from the TCGA database. High expression of SASH3 was not only linked to poor OS in ESCC, LAML, LGG, and UVM, but also associated with better OS in CESC, HNSC, LUAD, SARC, SKCM, THYM, and UCEC. As for DSS, a high level of SASH3 correlated with adverse DSS in ESCC, LGG, and UVM, and lowly expressed SASH3 was associated with shorter OS in CESC, HNSC, LUAD, SARC, SKCM, and UCEC. The results of Cox regression and nomogram analyses confirmed that SASH3 was an independent factor for LUAD prognosis. Gene Ontology (GO), the Kyoto Encyclopedia of Genes and Genomes (KEGG), and gene set enrichment analysis (GSEA) results showed that SASH3 was involved in natural killer cell-mediated cytotoxicity, Th17 cell differentiation, PD-L1 expression and PD-1 checkpoint pathway in cancer, NF-kappa B signaling pathway, B-cell receptor signaling pathway, and Toll-like receptor signaling pathway. SASH3 expression was correlated with TMB in 28 cancer types and associated with MSI in 22 cancer types, while there was a negative correlation between SASH3 expression and DNA methylation in diverse human cancer. The high DNA methylation level of SASH3 was correlated with better OS in KIRC and UVM, and associated with poor OS in SKCM. Moreover, we uncover that SASH3 expression was positively associated with the stroma score in 27 cancer types, the microenvironment score, and immune score in 32 cancer types, 38 types of immune cells in 32 cancer types, the 45 immune stimulators, 24 immune inhibitors, 41 chemokines, 18 receptors, and 21 major histocompatibility complex (MHC) molecules in 33 cancer types. Finally, forced SASH3 expression inhibited lung adenocarcinoma (LUAD) cell proliferation and cell migration. Our findings confirmed that SASH3 may be a biomarker for the prognosis and diagnosis of human cancer.

## Introduction

Cancer is a major cause of death worldwide and results in considerable social and economic burdens; breast, lung, and liver cancer are the main causes of high mortality worldwide (1). Despite improvements in the diagnosis and treatment of cancer, the prevalence of cure is low (2). Tumor immunotherapy has greatly improved the clinical outcomes of cancer patients, but many patients remain insensitive to immunotherapy (3). Therefore, it is urgent to determine the underlying molecular mechanism of cancer progression and identification of specific and sensitive biomarkers for the diagnosis and treatment of cancer.

The SAM and SH3 domain-containing protein family includes SASH1, SAMSN1, and SASH3 (4). It has been shown that SAM and SH3 domain-containing 1 (SASH1) is a putative tumor suppressor gene in lung, breast, thyroid and colorectal cancers (5). Emerging evidence has demonstrated that downregulation of SASH1 was associated with adverse clinical outcomes in colon cancer and glioma (6, 7). Numerous studies have demonstrated that SASH1 inhibited cancer cell growth, EMT, cell migration, and invasive cell behavior in hepatocarcinoma, thyroid, and cervical cancer cell lines (8, 9). Recent studies found that promoter DNA hypermethylation led to SASH1 repression in tumors (10). SH3 domain and nuclear localization signals 1 (SAMSN1) was found to encode one of a family of SH3 domain-containing cytoplasmic adaptor proteins expressed in lymphocytes (11). It has been reported that SAMSN1 was elevated and correlated with adverse clinical outcomes in glioblastoma multiforme (GBM) (11). On the contrary, Kodera et al. found that SAMSN1 was downregulated in hepatocellular carcinoma (HCC) and related to the malignant phenotype of HCC; multivariate analysis confirmed SAMSN1 as an independent prognostic factor of HCC progression (12). SASH3, also called SH3-containing lymphocyte protein (SLY1), was found be play an important role in the signal transduction cascades in lymphocytes. Recent studies have demonstrated that SASH3 was downregulated in breast cancer and correlated with a good prognosis (13). Furthermore, a recent study found that SASH3 mutation significantly reduced the CD4+ T-cell lymphopenia and promotes T-cell apoptosis in response to mitogens (14). Notarangelo et al. found that depletion of SASH3 results in immunodeficiency and immune dysregulation in human lymphocyte (14). However, the immunological function of SASH3 in pan-cancer is insufficient.

This study comprehensively analyzed the role of SASH3 in pan-cancer, including expression level, clinical features, prognostic values, DNA methylation, and mutation status of SASH3. Moreover, we analyzed the association between SASH3 expression and TMB, MSI, tumor microenvironment (TME), immune cell infiltration, and immune-related gene expression in human cancer. Finally, the growth curve, transwell, and flow cytometry were used to determine the biological function of SASH3 on lung adenocarcinoma (LUAD) cells.

## Materials and Methods

### Data Acquisition and Processing

In order to examine the expression level and prognosis values of SASH3 in pan-cancer, we downloaded the expression data and clinical data from the TCGA website (https://portal.gdc.cancer.gov/repository) and the Genotype-Tissue Expression (GTEx) database. The gene expression profiles were normalized using the scale method provided in the “limma” R package. Data analysis was performed with the R (version 3.6.3) and ggplot2 [3.3.3] packages. The expression data were normalized to transcripts per kilobase million (TPM) values before further analysis. Moreover, the ROC curve was used to evaluate the diagnostic value of NCAPG L using the pROC R package and ggplot2 R package.

### Gene Set Enrichment Analysis

We utilized the GSEA software to analyze the potential signaling pathway and molecular function in LUAD (15, 16). A customized Perl script was used to perform GSEA between the high-SASH3 and low-SASH3 groups. According to the default statistical methods, an adjusted *p*-value < 0.05 was considered significant.

### UALCAN

UALCAN is an interactive web portal where in-depth analyses of TCGA gene expression data can be performed. In this study, we used the UALCAN portal (http://ualcan.path.uab.edu) to examine the protein level of SASH in pan-cancer (17).

### cBioPortal and GeneMANIA

The website of cBioPortal (https://www.cbioportal.org/) offers an overview and detailed mutation-related information of SASH3. In this study, we used the cBioPortal database to determine the mutation feature of SASH3 in human cancer. The GeneMANIA (http://genemania.org/) prediction server: biological network integration for gene prioritization and predicting gene function. In this study, we employed GeneMANIA (http://genemania.org/) to analyze the interaction gene with the SASH3 in LUAD (18).

### GCSA

Gene Set Cancer Analysis (GSCA) is an enhanced version of GSCALite, which is a database used to search, investigate, and explore the gene set cancer analysis related to mRNA expression, mutation, immune infiltration, and drug resistance. In this study, we obtained the DNA methylation level of SASH3 provided by the GCSA website (http://bioinfo.life.hust.edu.cn/GSCA/#/) (19) and analyzed the correlation between DNA methylation and SASH3 expression in human cancer. Moreover, we determined the relationship between SASH3 DNA methylation level and the prognosis values.

### Analysis of the Immunological Roles of SASH3 in Pan-Cancer

We employed the TIMER (https://cistrome.shinyapps.io/timer/) and XCELL tools (https://xcell.ucsf.edu/) to analyze the immunological roles of SASH3, including the correlation between diverse immune cells and the immune regulator (15, 16). The TISIDB (http://cis.hku.hk/TISIDB/) was adopted to analyze the correlation between SASH3 expression and the diverse immune modulator in pan-cancer (20). The TMB and MSI scores were obtained from TCGA.

### Analysis of the Correlation Between SASH3 Expression and Drug Sensitivity

We employed the Genomics of Drug Sensitivity in Cancer (GDSC) (www.cancerRxgene.org) and The Cancer Therapeutics Response Portal (CTRP) (http://portals.broadinstitute.org/ctrp/) databases to analyze the correlation between SASH3 expression and drug sensitivity (21, 22).

### Cancer Cells and Cell Culture Conditions

The human bronchial epithelial (BEAS2B) cell line and LUAD cell lines were purchased from the cell bank of Kunming Institute of Zoology and cultured in BEGM media (Lonza, CC-3170). HEK-293T was obtained from ATCC. Lung cancer cell lines, including A549, HCC827, H1650, and H1975, were purchased from Cobioer, China with STR document, and A549, H1299, and H1975 cells were all cultured in RPMI 1640 medium (Corning) supplemented with 10% fetal bovine serum (Cat# 10099141C, Gibco, USA) and 1% penicillin/streptomycin. HEK-293T cells were cultured in DMEM medium (Corning).

### Constructs, Lenti-Viral Preparation, and Establishment of Different Cell Lines

Human SASH3 full-length cDNA was synthesized (Shanghai Generay Biotech) and sub-cloned into pCDH-CMV-E2F-eGFP lenti-viral vector. Lenti-viruses were generated according to the manufacturer’s protocol as previously documented (23), and indicated cells were infected by viruses twice with 48 h and 72 h viral supernatants containing 4 μg/ml polybrene, and stable cell lines were established by appropriate puromycin selection.

### Quantitative Real-Time PCR

The qRT-PCR assay was performed as documented (23). The primer sequences are listed as follows: SASH3-F: GTGATTTCCCGAACCATGAACA, SASH3-R: T TCCTCCAGAGTGTCTGCCATC; β-actin-F: CTTCGCGGGCGACGAT, β-actin-R: CCATAGGAATCCTTCTGACC. The expression quantification was obtained with the 2^−ΔΔCt^ method.

### Cell Proliferation Assay and Cell Migration Assay

Cell proliferation assay and cell migration assay were performed as previously described (24). Cell proliferation assay and cell migration assay were conducted to explore the biological function of SASH3 on LUAD cells.

### Statistical Analysis

For the datasets from the TCGA database, statistical analyses were performed using R (v.3.6.3). The Kaplan–Meier (KM) method was used to calculate cancer patient survival rates. Univariate and multivariate Cox analyses were performed to assess the correlation between clinical features and overall survival (OS), disease-specific survival (DSS), and progression-free survival (PFS). For the data regarding the function of SASH3, GraphPad Prism 7.0 was used for statistical analyses. The Student’s *t*-test evaluated the statistical significance between groups. The significance of the data between two experimental groups was determined by Student’s *t*-test, and multiple group comparisons were analyzed by one-way ANOVA. *p* < 0.05 (*), *p* < 0.01 (**), and *p* < 0.001 (***) were significant.

## Results

### SASH3 Expressed Differentially Between Tumor and Normal Tissues

**Figure 1:**
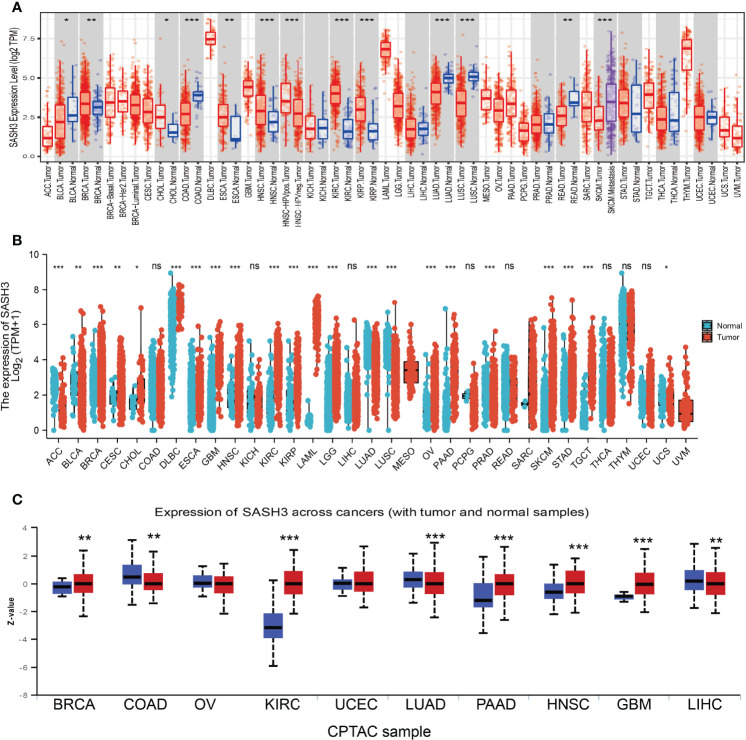
SASH3 expressed differentially between tumor and normal tissues. **(A)** The expression of SASH3 in pan-cancer analysis by the TIMER database. **(B)** The expression of SASH3 in pan-cancer analysis by the TCGA/GTEx database. **(C)** The protein of SASH3 in pan-cancer analysis by the UALCAN database. ACC, adrenocortical carcinoma; BLCA, bladder urothelial carcinoma; BRCA, breast invasive carcinoma; CESC, cervical squamous cell carcinoma and endocervical adenocarcinoma; CHOL, cholangiocarcinoma; COAD, colon adenocarcinoma; DLBC, lymphoid neoplasm diffuse large B-cell lymphoma; ESCA, esophageal carcinoma; GBM, glioblastoma multiforme; HNSC, head and neck squamous cell carcinoma; KICH, kidney chromophobe; KIRC, kidney renal clear cell carcinoma; KIRP, kidney renal papillary cell carcinoma; LAML, acute myeloid leukemia; LIHC, liver hepatocellular carcinoma; LUAD, lung adenocarcinoma; LUSC, lung squamous cell carcinoma; MESO, mesothelioma; OV, ovarian serous cystadenocarcinoma; PAAD, pancreatic adenocarcinoma; PCPG, pheochromocytoma and paraganglioma; PRAD, prostate adenocarcinoma; READ, rectum adenocarcinoma; SARC, sarcoma; SKCM, skin cutaneous melanoma; STAD, stomach adenocarcinoma; TGCT, testicular germ cell tumors; THCA, thyroid carcinoma; THYM, thymoma; UCEC, uterine corpus endometrial carcinoma; UCS, uterine carcinosarcoma; UVM, uveal melanoma. ns, *p* > 0.05, *p < 0.05, **p < 0.01, ***p < 0.001.

First, we examined the expression level of SASH3 in human cancers in the TCGA cohort with the corresponding normal tissue as control. We found that SASH3 expression was increased in breast invasive carcinoma (BRCA), cholangiocarcinoma (CHOL), head and neck squamous cell carcinoma (HNSC), kidney renal clear cell carcinoma (KIRC), and kidney renal papillary cell carcinoma (KIRP) tissues compared with adjacent normal tissues. Meanwhile, low expression of SASH3 in cancer was observed in bladder urothelial carcinoma (BLCA), colon adenocarcinoma (COAD), esophageal carcinoma (ESCA), LUAD, lung squamous cell carcinoma (LUSC), and rectum adenocarcinoma (READ) (**Figure 1A**). Because some cancers lack corresponding normal tissue controls, we therefore combined the data from the TCGA and the GTEx database, and the results confirmed that SASH3 expression was significantly higher in adrenocortical carcinoma (ACC), BRCA, CHOL, esophageal carcinoma (ESCA), GBM, HNSC, KIRC, KIRP, acute myeloid leukemia (LAML), brain lower-grade glioma (LGG), ovarian serous cystadenocarcinoma (OV), pancreatic adenocarcinoma (PAAD), READ, skin cutaneous melanoma (SKCM), stomach adenocarcinoma (STAD), testicular germ cell tumors (TGCT), and uterine carcinosarcoma (UCS) than in paired adjacent normal tissues (**Figure 1B**). To investigate the protein level of SASH3 in human cancer, we used UALCAN database analysis and proved that SASH3 was highly expressed in BRCA, KIRC, PAAD, HNSC, and GBM. Additionally, we also found that SASH3 was downregulated in COAD, LUAD, and testicular germ cell tumors (LIHC) (**Figure 1C**).

### Prognostic Values of SASH3 in Human Pan-Cancer

**Figure 2:**
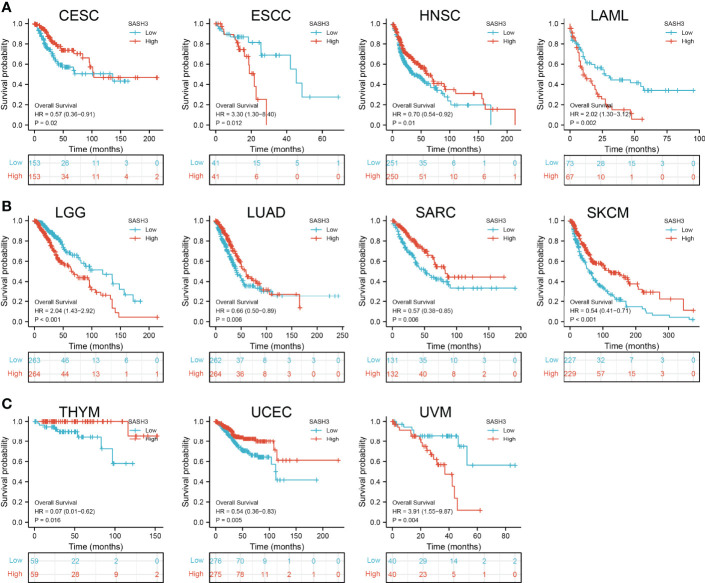
SASH3 expression correlated with the overall survival of pan-cancer. **(A)** The overall survival for SASH3 in CESC, ESCC, HNSC, and LAML. **(B)** The overall survival for SASH3 in LGG, LUAD, SARC, and SKCM. **(C)** The overall survival for SASH3 in THYM, UCEC, and UVM.

**Figure 3:**
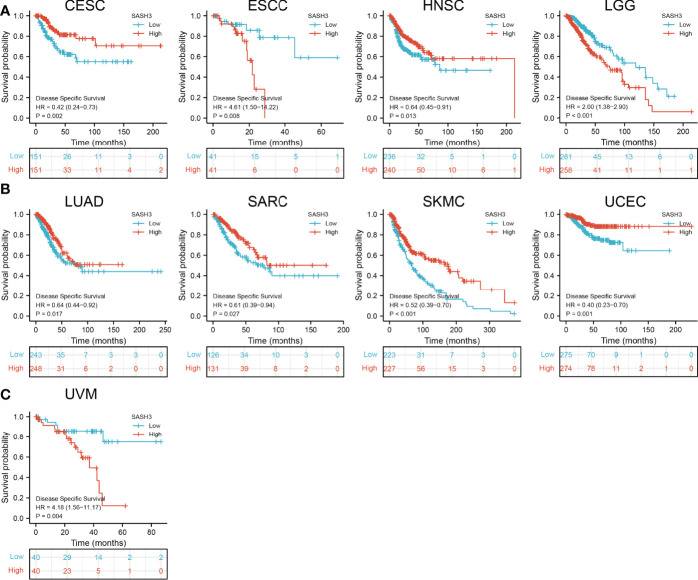
SASH3 expression correlated with the disease-specific survival of pan-cancer. **(A)** The disease-specific survival for SASH3 in CESC, ESCC, HNSC, and LGG. **(B)** The disease-specific survival for SASH3 in LUAD, SARC, UCEC, and SKCM. **(C)** The disease-specific survival for SASH3 in UVM.

**Figure 4:**
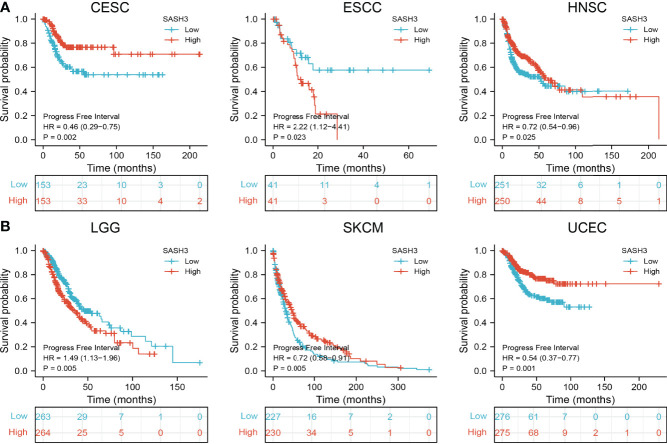
SASH3 expression correlated with the disease-free survival of pan-cancer. **(A)** The disease-free survival for SASH3 in CESC, ESCC, and HNSC. **(B)** The disease-free survival for SASH3 in LGG, SKCM, and UCEC.

To ascertain the prognostic role of SASH3 in different types of cancer, we ascertained the OS, DSS, and relapse-free survival (RFS) in human cancer types. KM survival curves demonstrated that higher SASH3 expression was not only linked to worse OS in ESCC, LAML, LGG, and UVM, but also associated with better OS in CESC, HNSC, LUAD, SARC, SKCM, THYM, and UCEC (**Figures 2A–C**). Results from the KM of DSS analysis suggested that increased SASH3 expression was correlated with adverse DSS in ESCC, LGG, and UVM; lowly expressed SASH3 was associated with shorter OS in CESC, HNSC, LUAD, SARC, SKCM, and UCEC (**Figures 3A–C**). Subsequently, we found that in CESC, HNSC, SKCM, and UCEC, patients with high SASH3 levels had longer PFS, and in ESCC and LGG, patients with low SASH3 levels had longer PFS (**Figures 4A, B**).

### SASH3 May Act as a Potential Biomarker in Human Cancer

**Figure 5:**
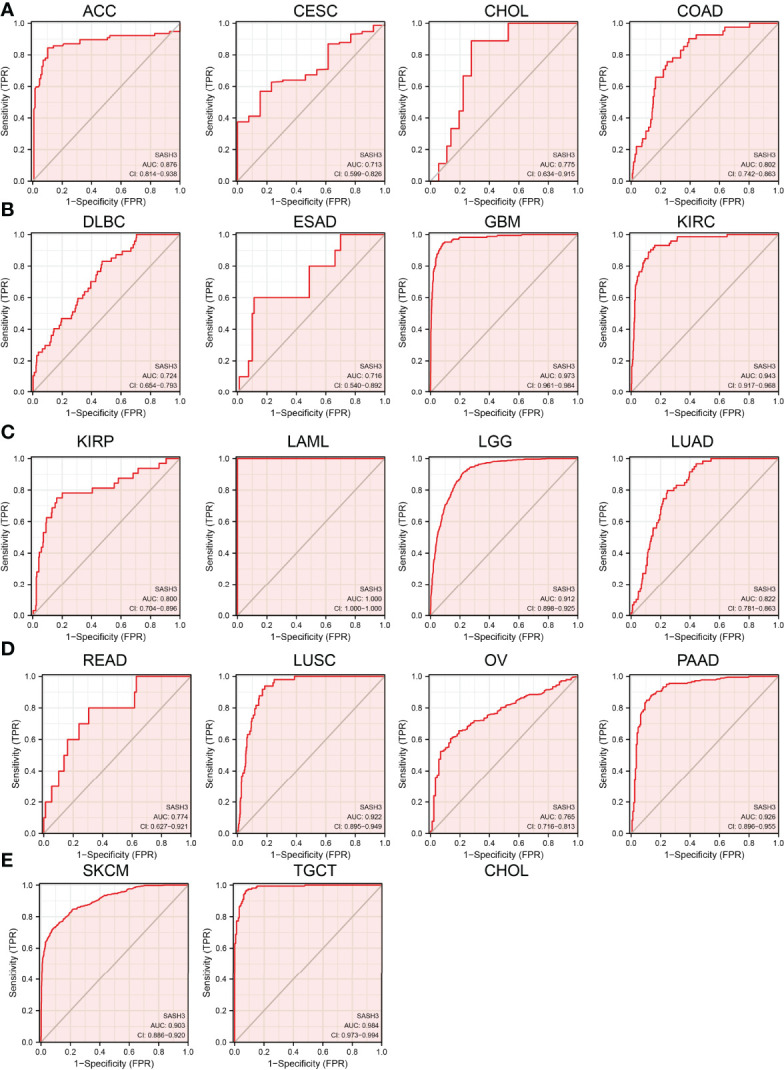
SASH3 may act as a potential biomarker in human cancer. Predictive power for prognosis with SASH3 expression by ROC curve analysis in ACC, CESC, CHOL, and COAD **(A)**; DLBC, ESAD, GBM, and KIRC **(B)**; KIRP, LAML, LGG, and LUAD **(C)**; READ, LUSC, OV, and PAAD **(D)**; SKCM and TGCT **(E)**.

We showed above that SASH3 expression was correlated with the prognosis of patients with different cancer types. Next, we investigated whether SASH3 could act as a biomarker for different cancer types. We conducted the ROC curve analysis and found that the AUC values for BRCA, PRAD, LUSC, LUAD, KIRP, KIRC, COAD, READ, THCA, GBM, LGG, PAAD, SKCM, LIHC, STAD, and ESCA are given in **Figures 5A–E**. Results confirmed that SASH3 could be used as a biomarker to diagnose different types of cancer with high sensitivity and specificity.

### Mutational Analysis of SASH3

**Figure 6:**
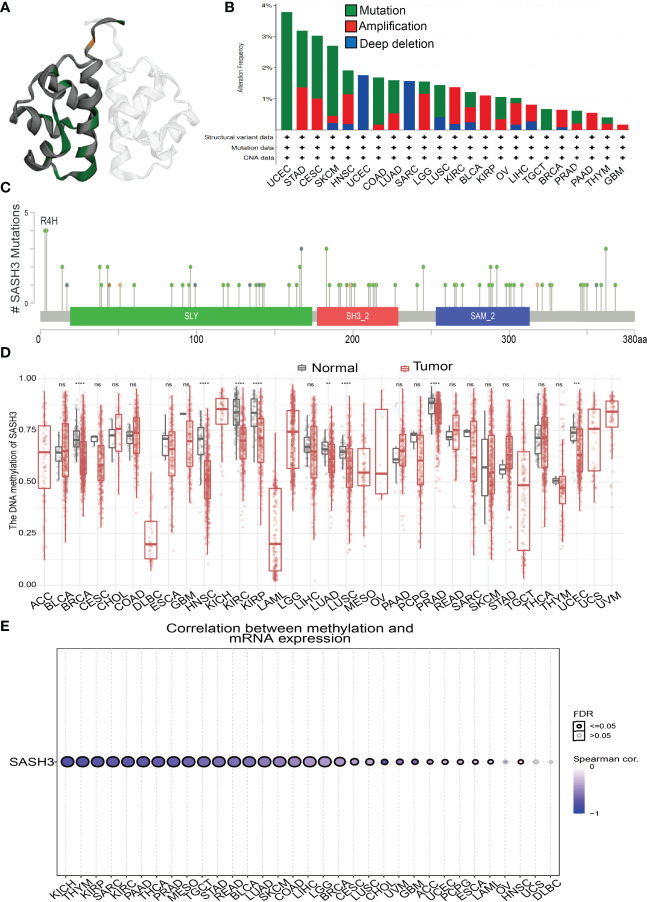
Mutational analysis of SASH3. **(A)** 3D structure of SASH3 gene transcript. **(B)** Summary of mutation types of SASH3 (structural variant data, mutation data, and copy number variant data) and the distribution among different cancers. **(C)** Hot spots of mutation of SASH3. **(D)** The DNA methylation level of SASH3 in pan-cancer. **(E)** Correlation between DNA methylation level and SASH3 expression in pan-cancer. **p < 0.01, ***p < 0.001, ****p < 0.00001, NS, p>0.05.

We download the mutational data of SASH3 from the cBioPortal database. According to the TCGA pan-cancer atlas, a three-dimensional image of the SASH3 protein was acquired (**Figure 6A**). Moreover, UCEC ranked first in SASH3 alteration frequency, followed by STAD and CESC (**Figure 6B**). The distribution of the mutation spot is shown in **Figure 6C**. A growing body of evidence confirmed that DNA methylation plays a central role in gene expression regulation and cancer progression (25). Therefore, we decided to investigate the potential association between DNA methylation and SASH3 expression. We utilized the GCSA database to explore the DNA methylation level of SASH3 in human cancer. Results showed that in cancers including BRCA, HNSC, KIRC, KIRP, LUAD, LUSC, PRAD, and UCEC, SASH3 was significantly differentially methylated between tumor and normal tissues (**Figure 6D**). We also demonstrate a negative correlation between DNA methylation and SASH3 expression in KICH, THYM, KIRP, SARC, PAAD, THCA, PRAD, MESO, TGCT, STAD, READ, BLCA, LUAD, SKCM, COAD, LIHC, LGG, and BRCA (**Figure 6E**).

Additionally, we found that in KIRC and UVM, the high DNA methylation of SASH3 was correlated with better OS, and associated with poor OS in SKCM (**Supplementary Figure 1**). For the DSS, the increased DNA methylation level of SASH3 was related to better DSS in KIRC, UVM, and LGG, and was associated with poor DSS in CESC (**Supplementary Figure 2**). For the PFS, the high DNA methylation level of SASH3 was correlated with poor PFS in BRCA, ACC, and LIHC, and related to better PFS in LGG (**Supplementary Figure 3**).

### GO and KEGG Analyses of SASH3 in Pan-Cancer

**Figure 7:**
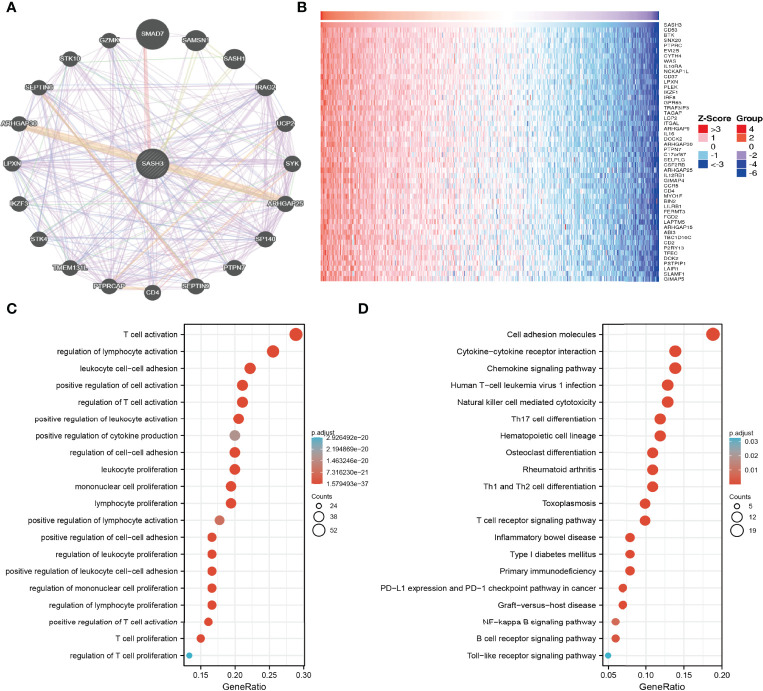
KEGG enrichment analysis. **(A)** The gene–gene interaction network of SASH3 examined by the GeneMANIA database. **(B)** The heatmap of the top 50 genes positively correlated with P4HA1 expression. **(C)** GO analysis of the biological process of SASH3 in pan-cancer. **(D)** KEGG pathway study revealed that these genes were involved in the different signaling pathways.

By using GeneMANIA, we generated the gene-to-gene network that included 20 potential interactors with SASH3, namely, SMAD7, SAMSN1, SASH1, IRAG2, UCP2, SYK, ARHGAP25, SP140, PTPN7, SEPTIN9, CD4, PTPRCAP, TME131L, STK4, IKZF3, LPXN, ARHGAP30, SEPTIN6, STK10, and GZMK (**Figure 7A**). Moreover, we identified genes with positive coexpression with SASH3 using the TCGA database, and the heatmap showed the top 100 genes that are positively correlated with SASH3 in pan-cancer (**Figure 7B** and **Supplementary Table 1**, *r* > 0.6, *p* < 0.0001). To determine the potential molecular mechanisms by which SASH3 modulates oncogenesis, we conducted the KEGG and GO enrichment using the 300 genes that were positively related to SASH3 in pan-cancers, respectively. As shown in **Figures 7C, D**, for the biological process, these genes were mainly enriched in T-cell activation, regulation of lymphocyte activation, leukocyte cell–cell adhesion, positive regulation of cell activation, regulation of T-cell activation, positive regulation of leukocyte activation, positive regulation of cytokine production, regulation of cell–cell adhesion, leukocyte proliferation, mononuclear cell proliferation, lymphocyte proliferation, positive regulation of lymphocyte activation, positive regulation of cell–cell adhesion, regulation of leukocyte proliferation, positive regulation of leukocyte cell–cell adhesion, regulation of mononuclear cell proliferation, regulation of lymphocyte proliferation, positive regulation of T-cell activation, T-cell proliferation, and regulation of T-cell proliferation (**Figure 7C**).

Moreover, KEGG pathway analysis suggested that SASH3 was associated with signaling pathways related to the cell adhesion molecules, Cytokine–cytokine receptor interaction, Chemokine signaling pathway, Human T-cell leukemia virus 1 infection, Natural killer cell-mediated cytotoxicity, Th17 cell differentiation, Hematopoietic cell lineage, Osteoclast differentiation, Rheumatoid arthritis, Th1 and Th2 cell differentiation, Toxoplasmosis, T-cell receptor signaling pathway, Inflammatory bowel disease, Type I diabetes mellitus, Primary immunodeficiency, PD-L1 expression and PD-1 checkpoint pathway in cancer, Graft-versus-host disease, NF-kappa B signaling pathway, B-cell receptor signaling pathway, and Toll-like receptor signaling pathway (**Figure 7D**).

### SASH3-Related Signaling Pathways in Cancers Identified by GSEA

**Figure 8:**
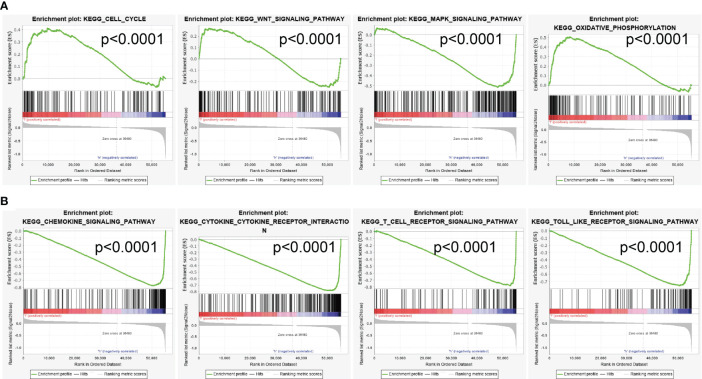
GSEA of SASH3. **(A, B)** The top GSEA results in pan-cancer.

To explore the potential signaling pathway of SASH3 involved in pan-cancer, the positive correlation between all protein-coding genes and SASH3 was calculated, and significant genes (*r* > 0.6, *p* < 0.001) were selected to perform gene set enrichment analysis (GSEA) (**Supplementary Table 1**). As is shown in **Figure 11**, GSEA results suggested that SASH3 mainly participated in cell cycle, Wnt signaling pathway, MAPK signaling pathway, oxidative phosphorylation, chemokine signaling pathway, cytokine–cytokine receptor interaction, T-cell receptor signaling pathway, and Toll-like receptor signaling pathway (**Figure 8**). These results confirmed that SASH3-regulated cell cycle and immune-related pathways might result in adverse survival of patients with cancer.

### Correlation Between SASH3 Expression and TMB and MSI

**Figure 9:**
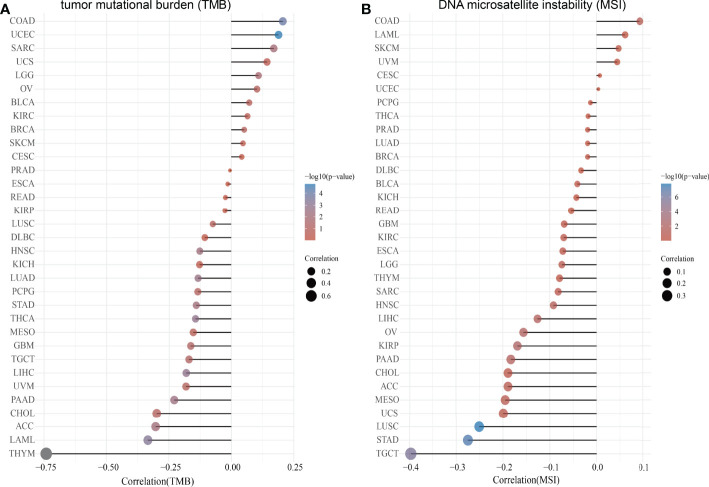
Correlation between SASH3 expression and TMB and MSI. **(A)** Analysis of the correlation between SASH3 and TMS in pan-cancer. **(B)** Analysis of the correlation between SASH3 and MSI in pan-cancer.

TMB is the number of non-inherited mutations per million bases of an investigated genomic sequence. TMB was reported to be a specific and sensitive biomarker of the response to immune checkpoint inhibitors (26). We examined the correlation between SASH3 expression and TMB of human cancers. SASH3 expression was markedly positively correlated with TMB in COAD, UCEC, SARC, UCS, LGG, OV, BLCA, KIRC, BRCA, SKCM, and CESC, and negatively correlated with TMB in DLBC, HNSC, KICH, LUAD, PCPG, STAD, THCA, MESO, GBM, TGCT, LIHC, UVM, PAAD, CHOL, ACC, LAML, and THYM (**Figure 9A**).

MSI represents a hyper-mutable state of DNA sequences caused by a lack of activity of DNA repair (27). We explored the correlation between SASH3 expression and MSI in human cancers. SASH3 expression was markedly positively correlated with MSI in COAD, LAML, SKCM, and UVM, and negatively correlated with MSI in GBM, KIRC, ESCA, LGG, THYM, SARC, HNSC, LIHC, OV, KIRP, PAAD, CHOL, ACC, MESO, UCS, LUSC, STAD, and TGCT (**Figure 9B**). Collectively, these data implied that SASH3 may influence antitumor immunity by regulating the composition and immune mechanism in the TME.

### Correlation Between SASH3 Expression and Immune Cell Infiltration in Pan-Cancer

**Figure 10:**
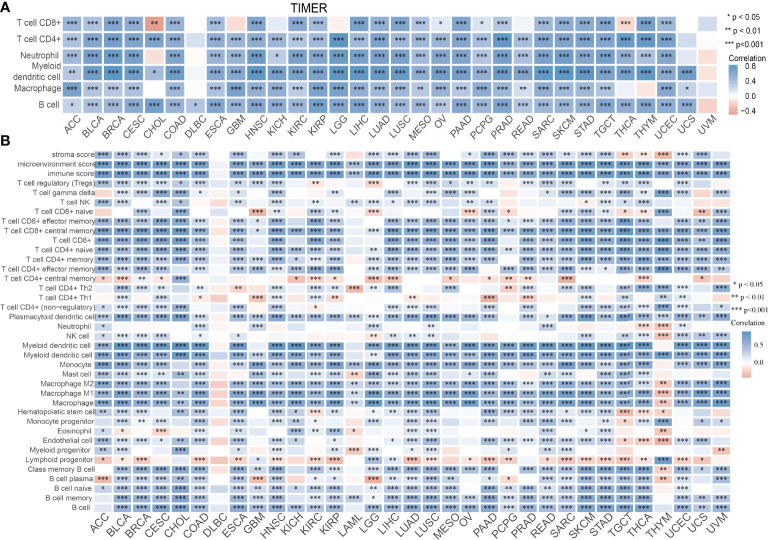
Correlation between SASH3 expression and immune infiltrates. **(A)** Correlations between SASH3 expression and the level of immune infiltration in 33 types of human cancer using TIMER. **(B)** Correlations between SASH3 expression and the level of immune infiltration in 33 types of human cancer using xCell. *p < 0.05, **p < 0.01, ***p < 0.001.

Infiltration of immune cells has an indispensable role in cancer progression (18); we then examined the relationship between SASH3 expression and the infiltration levels of T cell CD8+, T cell CD4+, Neutrophil, Myeloid dendritic cell, Macrophage, and B cell in 32 types of cancers using the TIMER database. We found that SASH3 expression was positively correlated with the infiltration levels of six major immune cells in 31 types of cancers (**Figure 10A**). To further confirm the correlation between SASH3 expression and infiltration of 38 subtypes of immune cell subtypes, by utilizing the xCell database, we found that SASH3 expression was correlated significantly with the stroma score in 27 cancer types, the microenvironment score, and the immune score in 32 cancer types, and 38 types of immune cells in 32 cancer types (**Figure 10B**). These findings indicated that SASH3 expression was significantly correlated with the infiltration of immune cells in human cancer.

### Correlation Between SASH3 Expression and Immune Modulators

**Figure 11:**
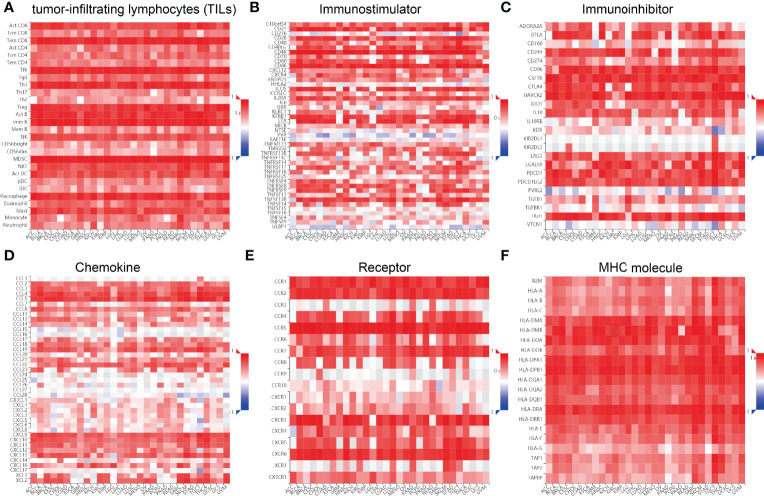
Correlation between SASH3 expression and immune modulators. Expression of SASH3 was significantly associated with tumor-infiltrating lymphocytes **(A)**, immune inhibitors **(B)**, immunostimulators **(C)**, chemokines **(D)**, receptors **(E)**, and MHCs **(F)**.

We wished to further understand the relationship between SASH3 and the TME. We examined the correlation between SASH3 expression and immune checkpoint-related genes using the TCGA database. SASH3 expression was positively correlated with immune checkpoint-related genes in 31 cancer types. These immune checkpoint-related genes were CD274, CTLA4, HAVCR2, LAG3, PDCD1, PDCD1LG2, SIGLEC15, and TIGIT (**Supplementary Figure 4**). Finally, we examine the correlation between SASH3 expression and the immune-related molecules. Our results confirmed that SASH3 expression was positively correlated with 28 tumor-infiltrating lymphocytes, 45 immune stimulators, 24 immune inhibitors, 41 chemokines, 18 receptors, and 21 major histocompatibility complex (MHC) molecules in different cancer types (**Figure 11**). These findings indicated that SASH3 had an indispensable role in regulation of the immune response in human cancer.

### Correlation Between SASH3 Expression and Drug Sensitivity

The results detailed above suggested that SASH3 may have roles in cancer progression. Next, we explored the correlation between SASH3 expression and drug response in different cancer cell lines from GDSC and CTRP. We demonstrate a negative correlation between SASH3 expression and diverse drugs, including I-BET-762, IK-93, PCA-1, A-793887, NPK76-II-72-1, Methotrexate, NG-25, KIN001-102, BX-912, CAL-101, XMD13-2, AT-7519, Tubastatin A, TG101348, JW-7-24-1, CP466722, BIX02189, and CAY10603 (**Supplementary Figure 5** and **Supplementary Table 2**, **3**, *r* < −0.4, *p* < 0.001). In summary, these results demonstrated that SASH3 expression was significantly negatively correlated with sensitivity to many drugs in different cancer cell lines.

### SASH3 Is an Independent Prognostic Factor in LUAD

**Figure 12:**
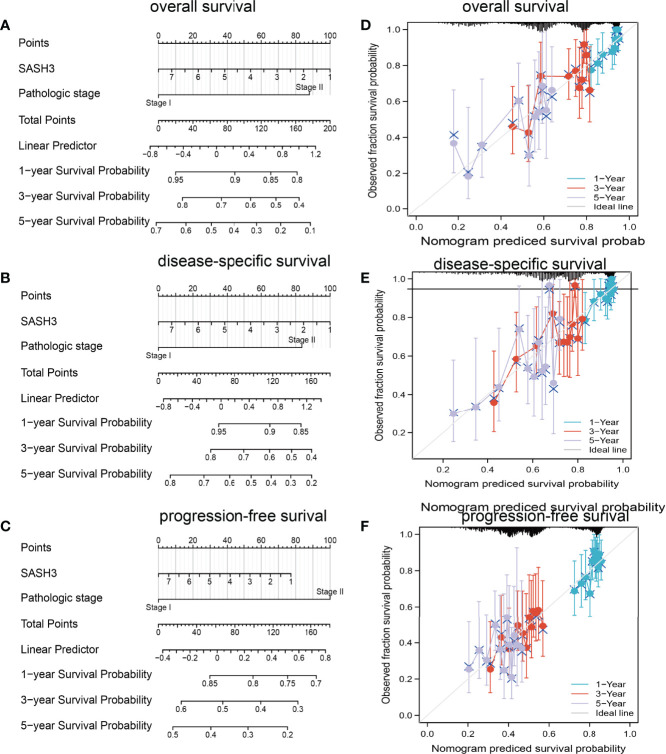
Construction and evaluation of nomogram. Nomogram to predict **(A)** overall survival, **(B)** disease-specific survival, and **(C)** progression-free survival of lung cancer patients. The calibration curve and Hosmer–Lemeshow test of nomograms in the TCGA-lung adenocarcinoma cohort for **(D)** overall survival, **(E)** disease-specific survival, and **(F)** progression-free survival.

To determine whether SASH3 expression level could be an independent prognostic factor for patients with LUAD, multivariate Cox regression analysis was performed. We showed that elevated SASH3 expression and pathological stage were a significant independent prognostic factor in the TCGA-LUAD cohort that directly correlated with poorer OS, DSS, and PFS outcomes (**Tables 1**–**3**). To examine the application of SASH3 in cancer prognosis, we built a nomogram for predicting the OS, DSS, and PFS of LUAD patients in the TCGA cohort. The pathological stage and SASH3 expression were included as prognostic factors in the nomogram (**Figures 12A–C**). The calibration curve showed that the nomogram was reliable in predicting the possibility of 1-, 3-, and 5-year OS in LUAD (**Figures 12D–F**). These results confirmed that the nomogram had better predictive power for the OS of LUAD patients, which might contribute to efficacy assessment and managing patients.

**Table 1:** Univariate and multivariate Cox regression analyses of different parameters on overall survival in LUAD.

Characteristics	Total (*N*)	Univariate analysis		Multivariate analysis	
		Hazard ratio (95% CI)	*p*-value	Hazard ratio (95% CI)	*p*-value
T stage	523				
T1 & T2	457				
T3 & T4	66	2.317 (1.591–3.375)	<0.001	1.714 (1.063–2.763)	0.027
N stage	510				
N0 & N1	437				
N3 & N2	73	2.321 (1.631–3.303)	<0.001	1.555 (0.758–3.189)	0.229
Pathologic stage	518				
Stage II & Stage I	411				
Stage IV & Stage III	107	2.664 (1.960–3.621)	<0.001	1.612 (0.751–3.460)	0.220
M stage	377				
M0	352				
M1	25	2.136 (1.248–3.653)	0.006	1.202 (0.550–2.627)	0.645
SASH3	526	0.840 (0.736–0.959)	0.010	0.852 (0.727–0.999)	0.048

**Table 2:** Univariate and multivariate Cox regression analyses of different parameters on disease specific survival in LUAD.

Characteristics	Total (*N*)	Univariate analysis		Multivariate analysis	
		Hazard ratio (95% CI)	*p*-value	Hazard ratio (95% CI)	*p*-value
T stage	488				
T1 & T2	430				
T3 & T4	58	1.974 (1.190–3.275)	0.008	1.472 (0.743–2.916)	0.268
N stage	475				
N0 & N1	410				
N3 & N2	65	1.971 (1.247–3.115)	0.004	1.432 (0.540–3.801)	0.470
Pathologic stage	483				
Stage II & Stage I	389				
Stage IV & Stage III	94	2.436 (1.645–3.605)	<0.001	1.486 (0.522–4.236)	0.458
M stage	344				
M0	323				
M1	21	2.455 (1.269–4.749)	0.008	1.574 (0.541–4.582)	0.405
SASH3	491	0.797 (0.675–0.941)	0.007	0.793 (0.650–0.969)	0.023

**Table 3:** Univariate and multivariate Cox regression analyses of different parameters on disease-free survival in LUAD.

Characteristics	Total (*N*)	Univariate analysis		Multivariate analysis	
		Hazard ratio (95% CI)	*p*-value	Hazard ratio (95% CI)	*p*-value
T stage	523				
T1 & T2	457				
T3 & T4	66	1.811 (1.249–2.628)	0.002	1.578 (1.049–2.374)	0.029
N stage	510				
N0 & N1	437				
N3 & N2	73	1.325 (0.914–1.919)	0.137		
Pathologic stage	518				
Stage II & Stage I	411				
Stage IV & Stage III	107	1.513 (1.105–2.071)	0.010	1.259 (0.888–1.785)	0.196
M stage	377				
M0	352				
M1	25	1.513 (0.855–2.676)	0.155		
SASH3	526	0.888 (0.785–1.004)	0.059	0.896 (0.789–1.017)	0.090

### SASH3 Inhibits Cancer Cell Growth, Cell Migration, and Induced Cell Cycle Arrest

**Figure 13:**
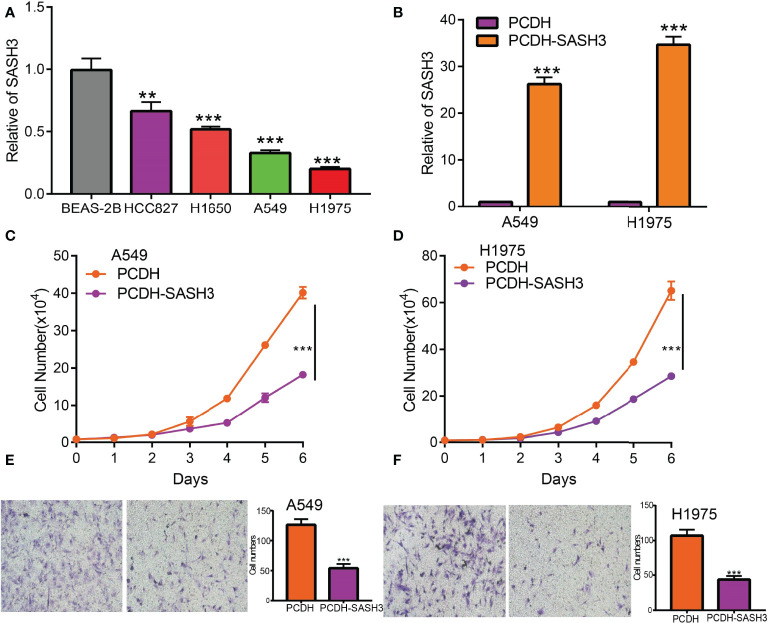
SASH3 inhibits cancer cell growth, cell migration, and induced cell cycle arrest. **(A)** The relative expression of SASH3 in LUAD cell lines including A549, HCC827, H1650, and H1975 examined by real-time RT-PCR; human bronchial epithelial (BEAS2B) cell line was used as control. β-actin was used as a housekeeping gene. **(B)** Establishment of SASH3 overexpression in A549 and H1975 cells, verified by real-time RT-PCR. **(C, D)** Indicated cell growth was examined by daily counting. **(E, F)** SASH3 overexpression inhibited A549 and H1975 cell migration by transwell assays. Quantification data were also indicated for each assay. **p < 0.01, ***p < 0.001.

Currently, there are still no studies that examine whether SASH3 is correlated with cancer progression. We decide to investigate the functional roles of SASH3 in LUAD. We found that SASH3 was decreased in the LUAD cell lines compared with the normal lung epithelial cell line (**Figure 13A**), which is consistent with the online database we discovered. Given that SASH3 was downregulated in LUAD, we then overexpressed the SASH3 using a lenti-viral system, and the overexpressed SASH3 mRNAs were verified by real-time RT-PCR assay (**Figure 13B**). We showed that overexpression of SASH3 inhibited tumor cell growth compared to the control cells (**Figures 13C, D**). To examine the potential role of SASH3 regulating tumor cell migration, we performed transwell assays. We found that forced expression of SASH3 inhibits cell migration, compared to the control group (**Figures 13E, F**). Together, these data suggest that SASH3 acts as a novel tumor suppressor in LUAD.

## Discussion

The treatment and prevention of cancer is a very important scientific research problem. Pan-cancer analysis is the crucial manner to compare the differences between diverse cancers and has great significant value for identifying new biomarkers and novel therapeutic targets of cancer (28). It has been confirmed that SASH3 was a potential prognostic factor for breast cancer patients (14). Currently, there is still no research that examines whether SASH3 is correlated with a cancer prognosis or can be a prognostic and diagnostic biomarker for human cancer.

In this study, we first analyzed SASH3 expression in diverse cancer. We found that SASH3 expression was the highest in ACC, BRCA, CHOL, ESCA, GBM, HNSC, KIRC, KIRP, LAML, LGG, OV, PAAD, READ, SKCM, STAD, TGCT, and UCS. On the contrary, we showed that SASH3 was downregulated in BLCA, COAD, ESCA, LUAD, LUSC, and READ.

In view of the clinical significance of SASH3 across different cancer types, we also examined whether SASH3 could be used as a potential biomarker for the early diagnosis of human cancers. For the prognostic and diagnostic value of SASH3 in pan-cancer, we found that high expression of SASH3 was not only linked to poor OS in ESCC, LAML, LGG, and UVM, but also associated with better OS in CESC, HNSC, LUAD, SARC, SKCM, THYM, and UCEC. As for DSS, a high level of SASH3 correlated with adverse DSS in ESCC, LGG, and UVM; lowly expressed SASH3 was associated with shorter OS in CESC, HNSC, LUAD, SARC, SKCM, and UCEC. However, in CESC, HNSC, SKCM, and UCEC, patients with high SASH3 levels had longer PFS, and in ESCC and LGG, patients with low SASH3 levels had longer PFS. ROC curve analysis showed that SASH3 could be used as a biomarker to diagnose different types of cancer with high sensitivity and specificity. The results of this study confirmed that SASH3 plays a critical role in cancer prognosis and may serve as an important prognostic and diagnostic biomarker.

Additionally, we discovered that mutation and methylation of SASH3 have a crucial influence on SASH3 mRNA expression. We found that UCEC patients had the highest alteration frequency of SASH3 (>3.5%). Additionally, we confirmed that alteration frequencies were 3.2%, 3%, 2.8%, and 1.9% in STAD, CESC, SKCM, and HNSC, respectively. DNA methylation dysregulation plays an indispensable role in the development and progression of cancer. We found a negative correlation between DNA methylation level and SASH3 expression in KICH, THYM, KIRP, SARC, PAAD, THCA, PRAD, MESO, TGCT, STAD, READ, BLCA, LUAD, SKCM, COAD, LIHC, LGG, and BRCA. Interestingly, SASH3 methylation was correlated with the prognosis of cancer patients, and we found that in KIRC and UVM, the high DNA methylation level of SASH3 was correlated with better OS, and associated with poor OS in SKCM. For the DSS, the increased DNA methylation level of SASH3 was related to better DSS in KIRC, UVM, and LGG, and was associated with poor DSS in CESC. For the PFS, the high DNA methylation level of SASH3 was correlated with poor PFS in BRCA, ACC, and LIHC, and related to better PFS in LGG.

The gene–gene interaction network was analyzed in the GeneMANIA database, and 20 proteins interacted with SASH3, namely, SMAD7, SAMSN1, SASH1, IRAG2, UCP2, SYK, ARHGAP25, SP140, PTPN7, SEPTIN9, CD4, PTPRCAP, TME131L, STK4, IKZF3, LPXN, ARHGAP30, SEPTIN6, STK10, and GZMK. At present, the potential molecular mechanism of SASH3 in tumors is only little understood. To further determine the potential molecular mechanism by which SASH3 affects cancer progression, we performed KEGG and GSEA analyses. Our KEGG enrichment analysis found that SASH3 may participate in Natural killer cell-mediated cytotoxicity, Th17 cell differentiation, Hematopoietic cell lineage, Osteoclast differentiation, Rheumatoid arthritis, Th1 and Th2 cell differentiation, Toxoplasmosis, T-cell receptor signaling pathway, Inflammatory bowel disease, Type I diabetes mellitus, Primary immunodeficiency, PD-L1 expression and PD-1 checkpoint pathway in cancer, Graft-versus-host disease, NF-kappa B signaling pathway, B-cell receptor signaling pathway, and Toll-like receptor signaling pathway.

The TME plays a significant role in cancer progression and even immune escape (20). Common immune cells included the neutrophils, natural killer cells, macrophages, dendritic cells, B cells, and T cells, and emerging evidence has demonstrated that immune cells play a critical role in the immune response and various cancer progression (24, 29). However, to date, there have been no studies on the function of SASH3 in the TME. Previous studies have shown that SASH3 was positively correlated with T cells in breast cancer (13). Some studies had shown that SASH3 was a potential prognostic factor for breast cancer patients and associated with the lymphocytic infiltrating tumor (13). For tumor immune cell infiltration, we found that SASH3 expression was positively correlated with the infiltration levels of T cell CD8+, T cell CD4+, Neutrophil, Myeloid dendritic cell, Macrophage, and B cell in 31 types of cancers. To further confirm the correlation between SASH3 expression and infiltration of 38 subtypes of immune cell subtypes, by utilizing the xCell database, we found that SASH3 expression was correlated significantly with the stroma score in 27 cancer types, the microenvironment score, and the immune score in 32 cancer types, and 38 types of immune cells in 32 cancer types. These findings indicated that SASH3 expression was significantly correlated with the infiltration of immune cells in human cancer. Given the effects of SASH3 on diverse human cancer immune cell infiltration, we can infer that increased expression of SASH3 may promote mast cell infiltration and contribute to a poor prognosis. Therefore, our results demonstrated that SASH3 might affect immune cell infiltration, making them a predictive biomarker for immunotherapy in cancer patients. Immune scores are usually utilized to evaluate the number of infiltrating immune cells in the TME. In this study, we found that the expression of SASH3 was correlated significantly with the stroma score in 27 cancer types, the microenvironment score, and immune score in 32 cancer types. This evidence indicated that SASH3 can regulate the immune cell infiltration and thus play a role in tumor regulation.

Recently, TMB and MSI have been highlighted as a immunotherapy biomarker for immune checkpoint inhibitors (30, 31). In our study, we discovered that SASH3 expression was markedly positively correlated with TMB in COAD, UCEC, SARC, UCS, LGG, OV, BLCA, KIRC, BRCA, SKCM, and CESC, and negatively correlated with TMB in DLBC, HNSC, KICH, LUAD, PCPG, STAD, THCA, MESO, GBM, TGCT, LIHC, UVM, PAAD, CHOL, ACC, LAML, and THYM. SASH3 expression was markedly positively correlated with MSI in COAD, LAML, SKCM, and UVM, and negatively correlated with MSI in GBM, KIRC, ESCA, LGG, THYM, SARC, HNSC, LIHC, OV, KIRP, PAAD, CHOL, ACC, MESO, UCS, LUSC, STAD, and TGCT. All of these lines of evidence confirmed for the first time that SASH3 plays an extremely crucial role in cancer progression and that SASH3 may be an important biomarker for the diagnosis, treatment, and prognosis of human cancer. Additionally, we conducted a correlation analysis between SASH3 expression and IC_50_ of 192 anticancer drugs and discovered that patients with low SASH3 expression might be resistant to most anti-cancer drugs, such as I-BET-762, IK-93, PCA-1, A-793887, NPK76-II-72-1, Methotrexate, NG-25, KIN001-102, BX-912, CAL-101, XMD13-2, AT-7519, and Tubastatin A.

Lung cancer is one of the main causes of cancer death, and it has brought huge public health burden worldwide (32). In our study, we found that increased SASH3 expression level and pathological stage were significant independent prognostic factors in the TCGA-LUAD cohort that directly correlated with poorer OS, DSS, and PFS outcomes. Furthermore, we built a nomogram for predicting the OS, DSS, and PFS of LUAD patients in the TCGA cohort. The pathological stage and SASH3 expression were included as prognostic factors in the nomogram. The calibration curve showed that the nomogram was reliable in predicting the possibility of 1-, 3-, and 5-year OS in LUAD. Currently, there are still no studies that examine whether SASH3 is correlated with cancer progression. We found that overexpression of SASH3 inhibited tumor cell growth and cell migration. Together, these data suggest that SASH3 acts as a novel tumor suppressor in LUAD.

This study improves our understanding of the correlation between SASH3 and human cancer, but some limitations still exist. First, although we explored the correlation between SASH3 and immune infiltration in pan-cancer patients, there is a lack of experiments to validate the function of SASH3 in the TME regulation of diverse cancer. Second, we uncover that forced SASH3 expression inhibits cell proliferation and cell migration of LUAD cells. However, the potential molecular mechanisms of SASH3 in tumor growth and metastasis need to be explored in further studies. Third, we did not conduct the *in vivo* experiments to validate the function of SASH3 in the tumor metastasis and TME regulation of LUAD.

## Conclusion

In conclusion, our finding discovered the potential function of SASH3 in pan-cancer for the first time. Additionally, we discovered that SASH3 expression was associated with prognosis, diagnosis, TMB, MSI, and infiltration levels of immunosuppressive cells in pan-cancer. SASH3 expression level was a significant independent prognostic factor in the TCGA-LUAD cohort that directly correlated with poorer OS, DSS, and PFS outcomes. Overexpression of SASH3 inhibited tumor cell growth and cell migration. Therefore, SASH3 can be used as a promising molecular predictor to evaluate the prognosis of cancer patients as well as a therapeutic target in the clinical detection of LUAD.

## Data Availability Statement

The original contributions presented in the study are included in the article/**Supplementary Material**. Further inquiries can be directed to the corresponding authors.

## Author Contributions

XC, YY, and WR designed this work and performed related assay. FZ and XH analyzed the data. JP, XN, and XJ supervised and wrote the manuscript. All authors contributed to the article and approved the submitted version.

## Funding

This study was supported by Applied Basic Research Project of Yunnan Provincial Science and Technology Department and Kunming Medical University, No. 2020001AY070001-117 and the Open Project of The First People’s Hospital of Yunnan Province Clinical Medicine Center (2021LCZXXF‐XZ03).

## Conflict of Interest

The authors declare that the research was conducted in the absence of any commercial or financial relationships that could be construed as a potential conflict of interest.

## Publisher’s Note

All claims expressed in this article are solely those of the authors and do not necessarily represent those of their affiliated organizations, or those of the publisher, the editors and the reviewers. Any product that may be evaluated in this article, or claim that may be made by its manufacturer, is not guaranteed or endorsed by the publisher.
