# Supplementary material for: Pan-Cancer Integrated Analysis Identification of SASH3, a Potential Biomarker That Inhibits Lung Adenocarcinoma Progression
Source: Front Oncol. 2022 Jun 3;12:927988. doi: 10.3389/fonc.2022.927988 (PMC9232268; doi:10.3389/fonc.2022.927988)
Supplement: Supplementary file 1 [file DataSheet_1.docx]

Supplementary Material

**
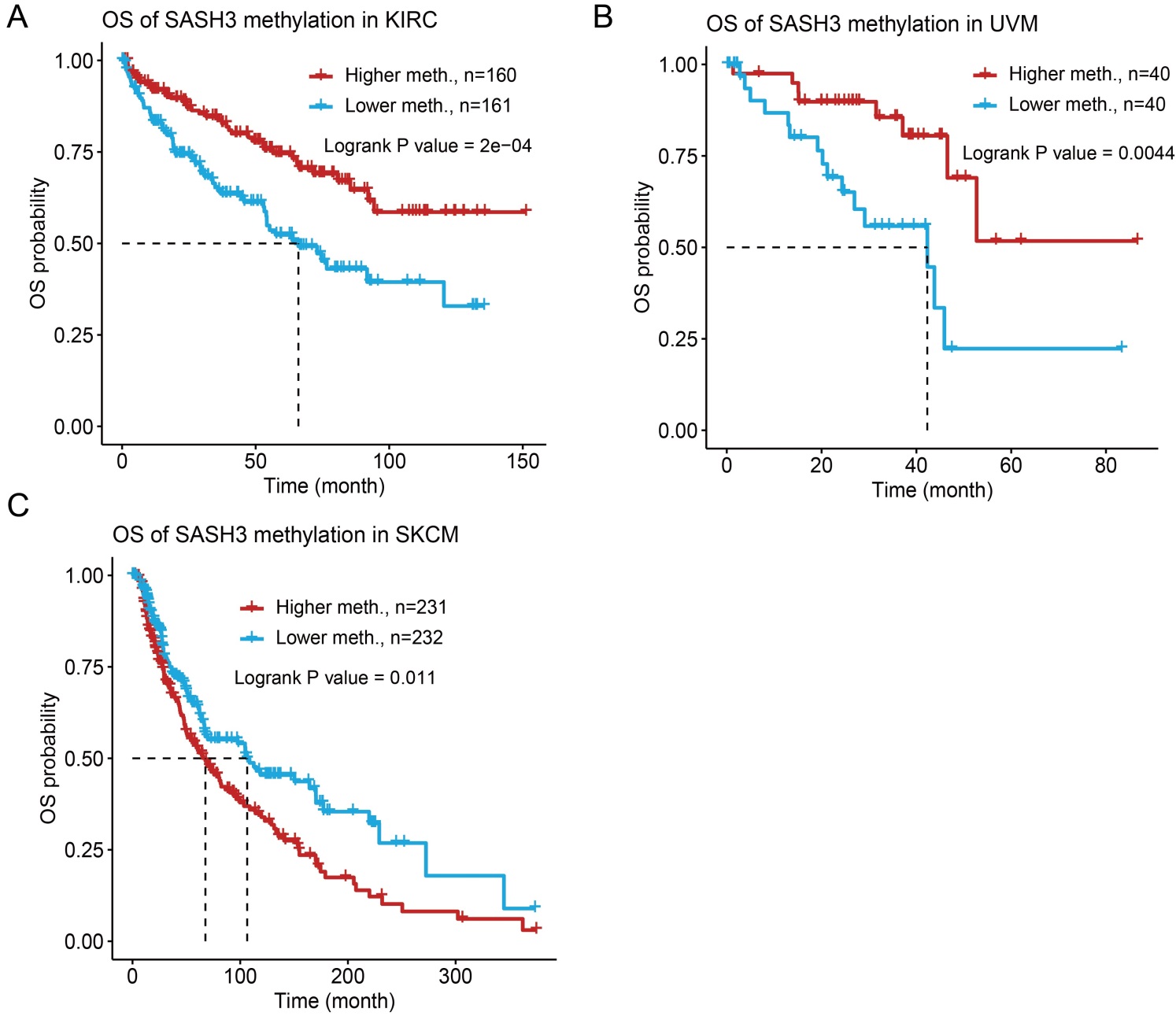
**

**Supplementary Figure 1. Correlation between methylation level of SASH3 and the overall survival patients with diverse cancer.**


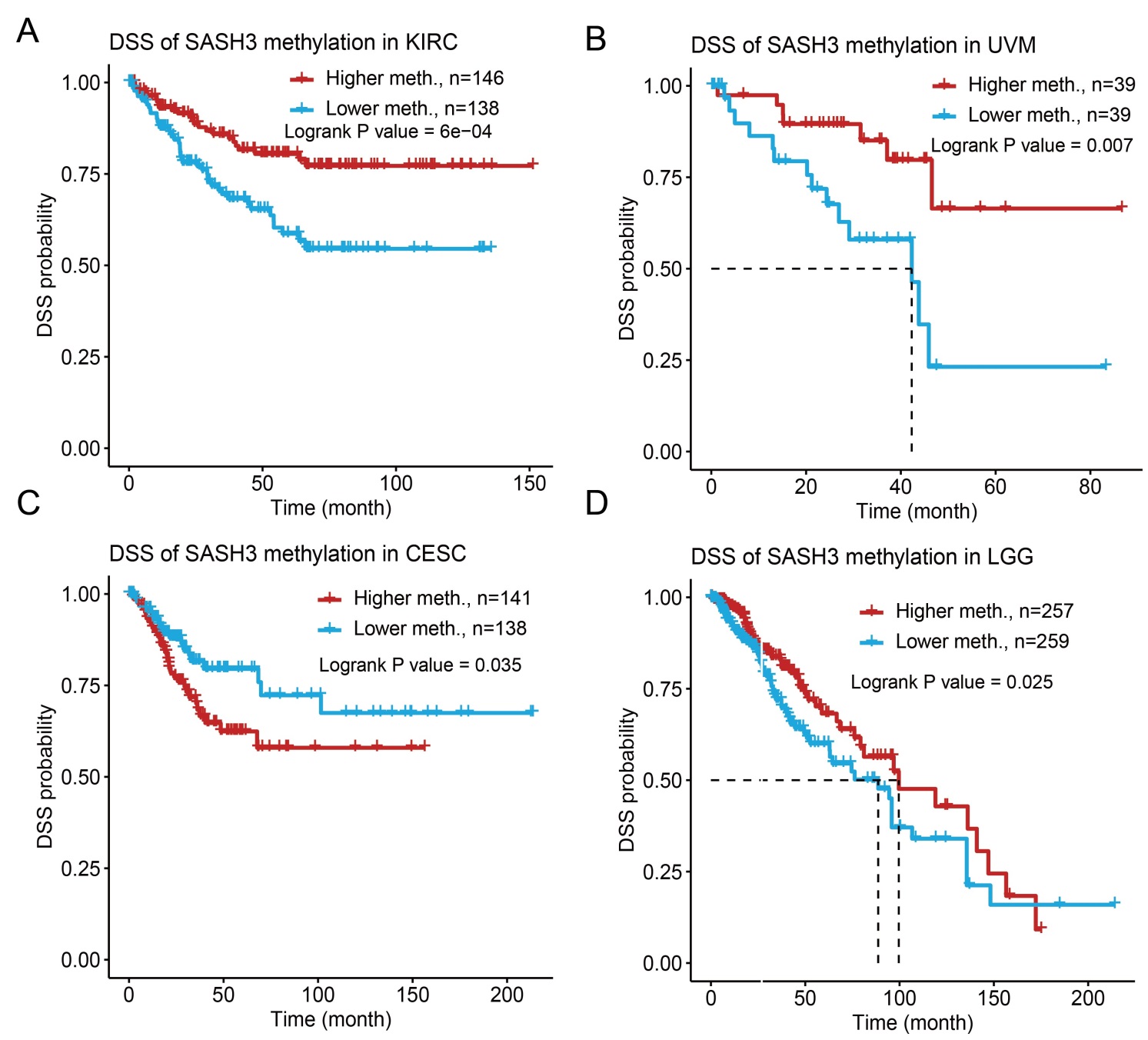


**Supplementary Figure 2. Correlation between methylation levels of SASH3 and the disease specific survival** **patients with diverse cancer.**

**
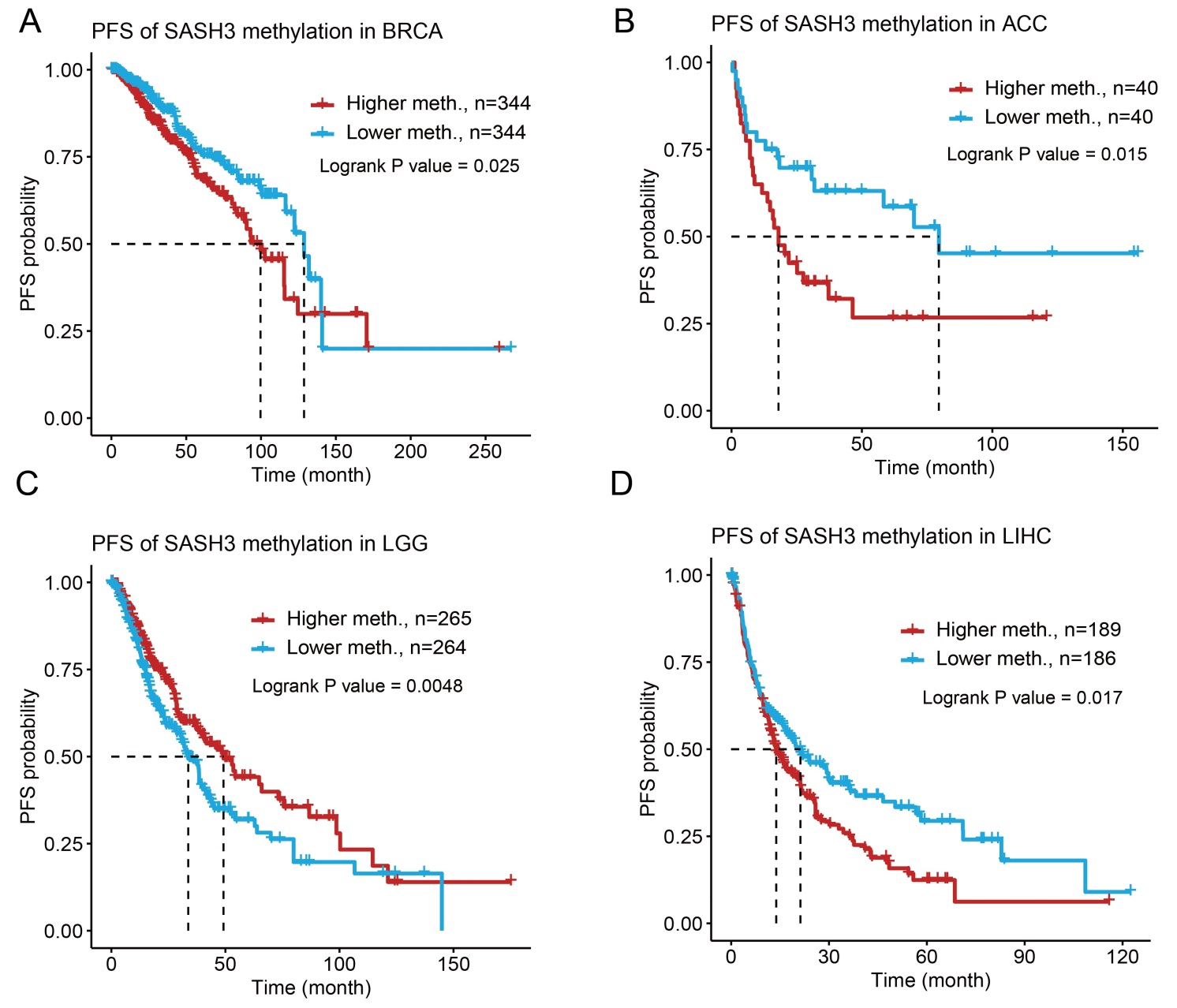
**

**Supplementary Figure 3. Correlation between methylation levels of SASH3 and the disease-free survival** **patients with diverse cancer.**


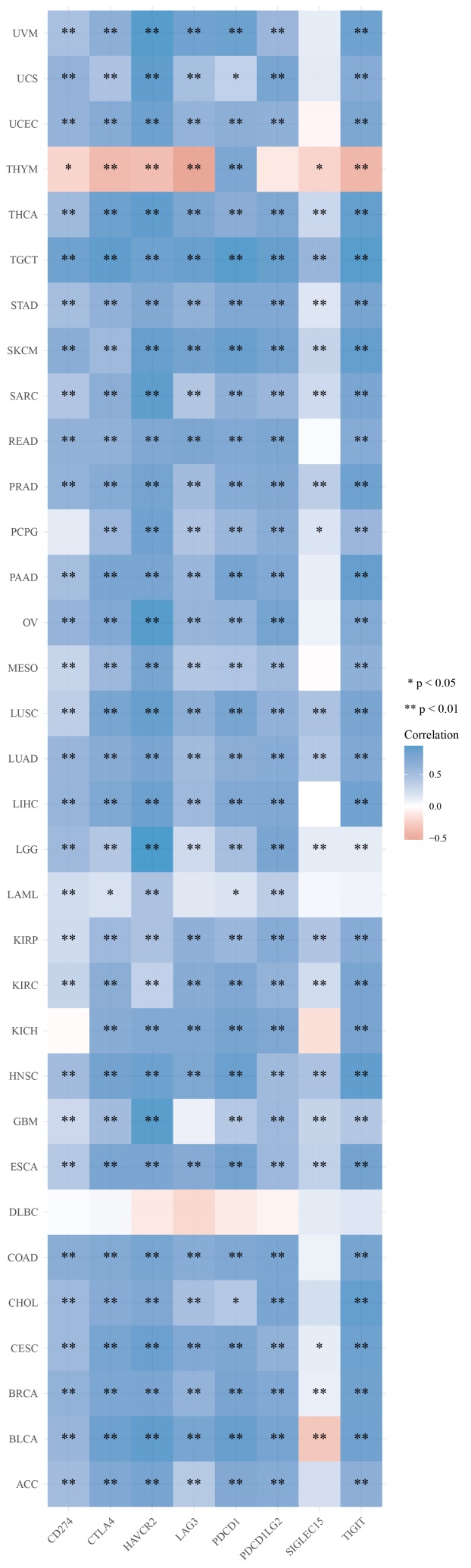


**Supplementary Figure 4. Analysis the correlation between the SASH3 expression and immune check points related gene. Analysis the correlation between the SASH3 expression and immune check points related gene in pan-cancer analysis by TIMER database.**


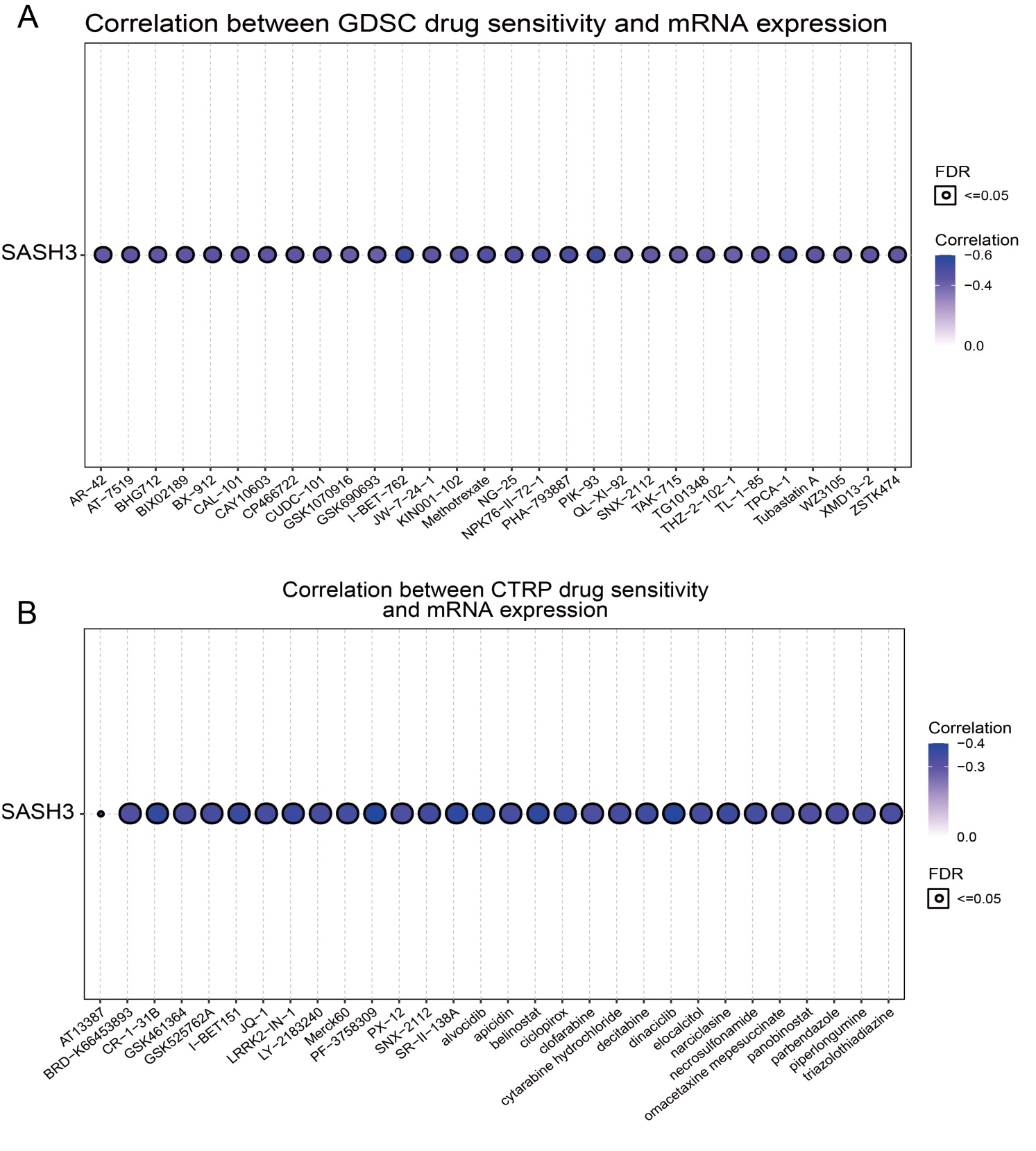


**Supplementary Figure 5. Figure 12 Analysis of the correlation between the SASH3 expression and Drug sensitivity in diverse human cancer.** (A) The correlation between the SASH3 expression and Drug sensitivity in diverse human cancer analysis by employed GDSC database. (B) The correlation between the SASH3 expression and Drug sensitivity in diverse human cancer analysis by employed CTRP database.

**Supplementary Table 1 The positive gene that correlation with SASH3 in pan-cancer**

| Query | Statistic | P-value | FDR (BH) |
| --- | --- | --- | --- |
| SASH3 | 1 | 1.00E-240 | 1.00E-236 |
| CD53 | 0.938916 | 8.36E-240 | 8.36E-236 |
| BTK | 0.938565 | 3.48E-239 | 2.32E-235 |
| SNX20 | 0.929369 | 3.58E-224 | 1.79E-220 |
| PTPRC | 0.928934 | 1.64E-223 | 6.55E-220 |
| EVI2B | 0.927372 | 3.51E-221 | 1.17E-217 |
| CYTH4 | 0.924347 | 8.28E-217 | 2.37E-213 |
| WAS | 0.923703 | 6.68E-216 | 1.67E-212 |
| IL10RA | 0.920119 | 5.40E-211 | 1.20E-207 |
| NCKAP1L | 0.919146 | 1.06E-209 | 2.12E-206 |
| CD37 | 0.915946 | 1.46E-205 | 2.66E-202 |
| LPXN | 0.913472 | 1.79E-202 | 2.99E-199 |
| PLEK | 0.912627 | 1.94E-201 | 2.98E-198 |
| IKZF1 | 0.910757 | 3.46E-199 | 4.94E-196 |
| IRF8 | 0.907619 | 1.61E-195 | 2.15E-192 |
| GPR65 | 0.907042 | 7.37E-195 | 9.20E-192 |
| TRAF3IP3 | 0.905815 | 1.81E-193 | 2.12E-190 |
| TAGAP | 0.904414 | 6.62E-192 | 7.35E-189 |
| LCP2 | 0.903422 | 8.17E-191 | 8.60E-188 |
| ITGAL | 0.895584 | 1.41E-182 | 1.41E-179 |
| ARHGAP9 | 0.89551 | 1.68E-182 | 1.60E-179 |
| IL16 | 0.892955 | 5.83E-180 | 5.30E-177 |
| DOCK2 | 0.891761 | 8.54E-179 | 7.42E-176 |
| ARHGAP30 | 0.891461 | 1.67E-178 | 1.39E-175 |
| PTPN7 | 0.891035 | 4.31E-178 | 3.45E-175 |
| C17orf87 | 0.889729 | 7.68E-177 | 5.90E-174 |
| SELPLG | 0.889001 | 3.76E-176 | 2.78E-173 |
| CSF2RB | 0.88883 | 5.46E-176 | 3.90E-173 |
| ARHGAP25 | 0.888489 | 1.14E-175 | 7.88E-173 |
| IL12RB1 | 0.887539 | 8.87E-175 | 5.91E-172 |
| GIMAP4 | 0.886049 | 2.12E-173 | 1.37E-170 |
| CCR5 | 0.885566 | 5.88E-173 | 3.67E-170 |
| CD4 | 0.885265 | 1.11E-172 | 6.72E-170 |
| MYO1F | 0.885213 | 1.24E-172 | 7.26E-170 |
| BIN2 | 0.884112 | 1.23E-171 | 7.03E-169 |
| LILRB1 | 0.883676 | 3.04E-171 | 1.69E-168 |
| FERMT3 | 0.883073 | 1.06E-170 | 5.71E-168 |
| FGD2 | 0.879471 | 1.56E-167 | 8.19E-165 |
| LAPTM5 | 0.875433 | 4.22E-164 | 2.16E-161 |
| ARHGAP15 | 0.874044 | 6.01E-163 | 3.00E-160 |
| ABI3 | 0.87236 | 1.44E-161 | 7.03E-159 |
| TBC1D10C | 0.869122 | 5.73E-159 | 2.73E-156 |
| CD2 | 0.868431 | 2.01E-158 | 9.36E-156 |
| P2RY13 | 0.86741 | 1.27E-157 | 5.79E-155 |
| TFEC | 0.866954 | 2.89E-157 | 1.28E-154 |
| DOK2 | 0.865221 | 6.31E-156 | 2.74E-153 |
| PSTPIP1 | 0.862968 | 3.26E-154 | 1.39E-151 |
| LAIR1 | 0.86125 | 6.29E-153 | 2.62E-150 |
| SLAMF1 | 0.861117 | 7.90E-153 | 3.22E-150 |
| GIMAP5 | 0.859134 | 2.29E-151 | 9.14E-149 |
| GMFG | 0.858657 | 5.10E-151 | 2.00E-148 |
| TNFAIP8L2 | 0.858437 | 7.37E-151 | 2.83E-148 |
| KIAA0748 | 0.858244 | 1.02E-150 | 3.84E-148 |
| AOAH | 0.857142 | 6.39E-150 | 2.36E-147 |
| FAM78A | 0.856114 | 3.49E-149 | 1.27E-146 |
| NCF1 | 0.854918 | 2.47E-148 | 8.83E-146 |
| RASAL3 | 0.854794 | 3.03E-148 | 1.06E-145 |
| MPEG1 | 0.854375 | 5.99E-148 | 2.06E-145 |
| SIT1 | 0.854169 | 8.37E-148 | 2.84E-145 |
| CCR2 | 0.853847 | 1.41E-147 | 4.70E-145 |
| CYBB | 0.853256 | 3.66E-147 | 1.20E-144 |
| CD180 | 0.851715 | 4.32E-146 | 1.39E-143 |
| ARHGEF6 | 0.850903 | 1.57E-145 | 4.98E-143 |
| ACAP1 | 0.850283 | 4.17E-145 | 1.30E-142 |
| SPN | 0.849896 | 7.67E-145 | 2.36E-142 |
| CD48 | 0.848729 | 4.76E-144 | 1.44E-141 |
| IL21R | 0.848285 | 9.50E-144 | 2.84E-141 |
| GIMAP7 | 0.847622 | 2.65E-143 | 7.80E-141 |
| LOC100233209 | 0.847025 | 6.67E-143 | 1.93E-140 |
| CD247 | 0.846935 | 7.65E-143 | 2.19E-140 |
| RCSD1 | 0.846036 | 3.04E-142 | 8.55E-140 |
| AIF1 | 0.84472 | 2.25E-141 | 6.24E-139 |
| AMICA1 | 0.844313 | 4.16E-141 | 1.14E-138 |
| PIK3R5 | 0.840997 | 5.86E-139 | 1.58E-136 |
| IGSF6 | 0.840374 | 1.47E-138 | 3.91E-136 |
| HAVCR2 | 0.840282 | 1.68E-138 | 4.42E-136 |
| MS4A6A | 0.839587 | 4.64E-138 | 1.21E-135 |
| SPI1 | 0.83863 | 1.87E-137 | 4.79E-135 |
| CORO1A | 0.838587 | 1.99E-137 | 5.04E-135 |
| PLEKHO2 | 0.838087 | 4.11E-137 | 1.03E-134 |
| SLA | 0.837939 | 5.09E-137 | 1.26E-134 |
| GIMAP1 | 0.837678 | 7.42E-137 | 1.81E-134 |
| SLAMF8 | 0.837384 | 1.13E-136 | 2.73E-134 |
| GIMAP6 | 0.837235 | 1.40E-136 | 3.34E-134 |
| SH2D1A | 0.836316 | 5.24E-136 | 1.23E-133 |
| CD52 | 0.835366 | 2.03E-135 | 4.71E-133 |
| CD28 | 0.835318 | 2.17E-135 | 4.98E-133 |
| LTA | 0.834684 | 5.32E-135 | 1.21E-132 |
| HCST | 0.834365 | 8.34E-135 | 1.87E-132 |
| KLHL6 | 0.83272 | 8.39E-134 | 1.86E-131 |
| EVI2A | 0.831733 | 3.31E-133 | 7.26E-131 |
| CD86 | 0.830876 | 1.08E-132 | 2.35E-130 |
| CD3E | 0.82969 | 5.50E-132 | 1.18E-129 |
| SLAMF6 | 0.829453 | 7.60E-132 | 1.62E-129 |
| CLEC10A | 0.828733 | 2.03E-131 | 4.27E-129 |
| PIK3AP1 | 0.827245 | 1.51E-130 | 3.15E-128 |
| TLR7 | 0.825096 | 2.68E-129 | 5.52E-127 |
| MNDA | 0.824196 | 8.81E-129 | 1.80E-126 |
| IL2RG | 0.823432 | 2.41E-128 | 4.87E-126 |
| NCF4 | 0.822842 | 5.22E-128 | 1.04E-125 |
| PARVG | 0.822469 | 8.50E-128 | 1.68E-125 |
| NFAM1 | 0.821548 | 2.82E-127 | 5.52E-125 |
| CD33 | 0.820567 | 1.00E-126 | 1.94E-124 |
| CRTAM | 0.820417 | 1.22E-126 | 2.34E-124 |
| PYHIN1 | 0.819702 | 3.05E-126 | 5.81E-124 |
| BTLA | 0.8196 | 3.48E-126 | 6.56E-124 |
| FGR | 0.818973 | 7.75E-126 | 1.45E-123 |
| ITK | 0.817386 | 5.83E-125 | 1.08E-122 |
| P2RY10 | 0.815866 | 3.95E-124 | 7.24E-122 |
| CD84 | 0.815369 | 7.35E-124 | 1.34E-121 |
| TLR8 | 0.814389 | 2.49E-123 | 4.49E-121 |
| ICOS | 0.8142 | 3.15E-123 | 5.63E-121 |
| CLECL1 | 0.814126 | 3.45E-123 | 6.11E-121 |
| C5orf20 | 0.813741 | 5.56E-123 | 9.76E-121 |
| MAP4K1 | 0.813401 | 8.47E-123 | 1.47E-120 |
| GVIN1 | 0.812407 | 2.88E-122 | 4.96E-120 |
| GAB3 | 0.809367 | 1.16E-120 | 1.98E-118 |
| CYTIP | 0.808575 | 3.00E-120 | 5.09E-118 |
| LCP1 | 0.808434 | 3.56E-120 | 5.98E-118 |
| SAMSN1 | 0.807974 | 6.17E-120 | 1.03E-117 |
| PRKCB | 0.807529 | 1.05E-119 | 1.74E-117 |
| FGL2 | 0.807151 | 1.65E-119 | 2.70E-117 |
| UBASH3A | 0.806495 | 3.59E-119 | 5.83E-117 |
| CD5 | 0.806462 | 3.73E-119 | 6.02E-117 |
| CMKLR1 | 0.805747 | 8.69E-119 | 1.39E-116 |
| C3AR1 | 0.804144 | 5.71E-118 | 9.05E-116 |
| APBB1IP | 0.802413 | 4.27E-117 | 6.72E-115 |
| HLA-DMB | 0.80224 | 5.22E-117 | 8.15E-115 |
| ITGB2 | 0.802118 | 6.00E-117 | 9.30E-115 |
| SAMD3 | 0.801981 | 7.04E-117 | 1.08E-114 |
| DOK3 | 0.801554 | 1.15E-116 | 1.76E-114 |
| LRRC25 | 0.801037 | 2.08E-116 | 3.16E-114 |
| TIFAB | 0.800519 | 3.78E-116 | 5.68E-114 |
| C1orf162 | 0.800266 | 5.04E-116 | 7.52E-114 |
| CD6 | 0.800061 | 6.37E-116 | 9.44E-114 |
| RNASE6 | 0.799848 | 8.13E-116 | 1.19E-113 |
| GPR18 | 0.79958 | 1.10E-115 | 1.61E-113 |
| HVCN1 | 0.798305 | 4.68E-115 | 6.78E-113 |
| CXorf21 | 0.798248 | 5.00E-115 | 7.19E-113 |
| GPR174 | 0.798067 | 6.13E-115 | 8.75E-113 |
| LCK | 0.798003 | 6.59E-115 | 9.34E-113 |
| THEMIS | 0.797989 | 6.69E-115 | 9.42E-113 |
| C1QA | 0.797888 | 7.50E-115 | 1.05E-112 |
| DOCK8 | 0.797769 | 8.58E-115 | 1.19E-112 |
| LY86 | 0.797695 | 9.33E-115 | 1.29E-112 |
| WIPF1 | 0.796941 | 2.18E-114 | 2.98E-112 |
| PIK3CG | 0.796536 | 3.42E-114 | 4.66E-112 |
| NCF1B | 0.795913 | 6.87E-114 | 9.28E-112 |
| CSF1R | 0.795607 | 9.66E-114 | 1.30E-111 |
| FCER1G | 0.793985 | 5.83E-113 | 7.77E-111 |
| PTPRCAP | 0.793775 | 7.35E-113 | 9.73E-111 |
| NCR3 | 0.793569 | 9.23E-113 | 1.21E-110 |
| TNFSF8 | 0.791907 | 5.70E-112 | 7.44E-110 |
| ITGB7 | 0.79183 | 6.20E-112 | 8.05E-110 |
| CD300LF | 0.7915 | 8.88E-112 | 1.15E-109 |
| TRPV2 | 0.791433 | 9.55E-112 | 1.22E-109 |
| C16orf54 | 0.790999 | 1.53E-111 | 1.95E-109 |
| NCF1C | 0.790573 | 2.43E-111 | 3.07E-109 |
| CD200R1 | 0.790002 | 4.50E-111 | 5.66E-109 |
| C1QB | 0.789928 | 4.88E-111 | 6.09E-109 |
| HCK | 0.788966 | 1.37E-110 | 1.71E-108 |
| HLA-DPB1 | 0.788737 | 1.76E-110 | 2.17E-108 |
| TRAT1 | 0.788064 | 3.61E-110 | 4.42E-108 |
| FLI1 | 0.787661 | 5.55E-110 | 6.76E-108 |
| TLR4 | 0.787499 | 6.59E-110 | 7.98E-108 |
| SIRPG | 0.78735 | 7.73E-110 | 9.30E-108 |
| 1-Mar | 0.787125 | 9.81E-110 | 1.17E-107 |
| SLCO2B1 | 0.786794 | 1.39E-109 | 1.66E-107 |
| CLEC4A | 0.786302 | 2.35E-109 | 2.78E-107 |
| GZMK | 0.785346 | 6.44E-109 | 7.57E-107 |
| RGS18 | 0.784608 | 1.40E-108 | 1.63E-106 |
| C1QC | 0.784169 | 2.22E-108 | 2.57E-106 |
| FYB | 0.783814 | 3.21E-108 | 3.71E-106 |
| FAIM3 | 0.783759 | 3.40E-108 | 3.90E-106 |
| KBTBD8 | 0.783272 | 5.65E-108 | 6.45E-106 |
| TIGIT | 0.783197 | 6.11E-108 | 6.94E-106 |
| ABCD2 | 0.783176 | 6.24E-108 | 7.05E-106 |
| HCLS1 | 0.782689 | 1.03E-107 | 1.16E-105 |
| SIGLEC9 | 0.782441 | 1.34E-107 | 1.49E-105 |
| SIGLEC7 | 0.782419 | 1.37E-107 | 1.52E-105 |
| 1-Sep | 0.782278 | 1.59E-107 | 1.75E-105 |
| TYROBP | 0.782177 | 1.76E-107 | 1.93E-105 |
| TNFSF13B | 0.782112 | 1.88E-107 | 2.06E-105 |
| LST1 | 0.781242 | 4.62E-107 | 5.02E-105 |
| SLC7A7 | 0.780961 | 6.17E-107 | 6.66E-105 |
| HLA-DRA | 0.780864 | 6.82E-107 | 7.32E-105 |
| ZNF831 | 0.780486 | 1.00E-106 | 1.07E-104 |
| ASB2 | 0.779743 | 2.15E-106 | 2.28E-104 |
| RAC2 | 0.779191 | 3.77E-106 | 3.99E-104 |
| NLRC3 | 0.779127 | 4.03E-106 | 4.24E-104 |
| CD226 | 0.779068 | 4.28E-106 | 4.48E-104 |
| CD3D | 0.778384 | 8.57E-106 | 8.93E-104 |
| SIGLEC10 | 0.777577 | 1.94E-105 | 2.01E-103 |
| FPR3 | 0.777046 | 3.32E-105 | 3.42E-103 |
| CXCR6 | 0.7751 | 2.33E-104 | 2.39E-102 |
| PIK3R6 | 0.77441 | 4.63E-104 | 4.72E-102 |
| HLA-DPA1 | 0.774115 | 6.21E-104 | 6.30E-102 |
| LSP1 | 0.774023 | 6.80E-104 | 6.87E-102 |
| LILRB4 | 0.77322 | 1.51E-103 | 1.51E-101 |
| GAPT | 0.772663 | 2.61E-103 | 2.61E-101 |
| PTPN22 | 0.772444 | 3.24E-103 | 3.22E-101 |
| SP140 | 0.772232 | 3.99E-103 | 3.95E-101 |
| MFNG | 0.771905 | 5.50E-103 | 5.41E-101 |
| CD80 | 0.771727 | 6.55E-103 | 6.42E-101 |
| KLRB1 | 0.771433 | 8.74E-103 | 8.52E-101 |
| STX11 | 0.771382 | 9.18E-103 | 8.90E-101 |
| GRAP2 | 0.771082 | 1.23E-102 | 1.19E-100 |
| FUT7 | 0.771045 | 1.28E-102 | 1.23E-100 |
| AGAP2 | 0.770984 | 1.35E-102 | 1.30E-100 |
| DPEP2 | 0.770359 | 2.49E-102 | 2.37E-100 |
| CD3G | 0.769878 | 3.98E-102 | 3.77E-100 |
| KCNAB2 | 0.768831 | 1.10E-101 | 1.03E-99 |
| APOL3 | 0.768006 | 2.43E-101 | 2.28E-99 |
| LILRB2 | 0.767001 | 6.37E-101 | 5.95E-99 |
| CD244 | 0.766786 | 7.82E-101 | 7.27E-99 |
| CD40LG | 0.766534 | 9.95E-101 | 9.21E-99 |
| TNFRSF1B | 0.766087 | 1.52E-100 | 1.40E-98 |
| GGTA1 | 0.765573 | 2.49E-100 | 2.28E-98 |
| CCR7 | 0.765524 | 2.60E-100 | 2.38E-98 |
| FCGR1A | 0.763965 | 1.14E-99 | 1.03E-97 |
| C10orf128 | 0.763882 | 1.23E-99 | 1.11E-97 |
| CR1 | 0.763228 | 2.28E-99 | 2.05E-97 |
| ITGAX | 0.762188 | 6.03E-99 | 5.41E-97 |
| CD1D | 0.762164 | 6.17E-99 | 5.50E-97 |
| SLC15A3 | 0.761029 | 1.78E-98 | 1.58E-96 |
| SELL | 0.760772 | 2.25E-98 | 1.99E-96 |
| GPR114 | 0.759749 | 5.81E-98 | 5.12E-96 |
| FCGR1C | 0.759657 | 6.32E-98 | 5.54E-96 |
| PILRA | 0.75938 | 8.17E-98 | 7.13E-96 |
| CIITA | 0.758525 | 1.79E-97 | 1.56E-95 |
| MIR155HG | 0.758329 | 2.15E-97 | 1.86E-95 |
| CD96 | 0.757859 | 3.30E-97 | 2.84E-95 |
| FCGR1B | 0.757427 | 4.90E-97 | 4.20E-95 |
| ZAP70 | 0.756564 | 1.07E-96 | 9.18E-95 |
| MS4A4A | 0.756506 | 1.13E-96 | 9.63E-95 |
| C13orf18 | 0.755152 | 3.86E-96 | 3.27E-94 |
| LOC100188949 | 0.754753 | 5.54E-96 | 4.67E-94 |
| HLA-DOA | 0.754624 | 6.21E-96 | 5.22E-94 |
| GIMAP2 | 0.754596 | 6.38E-96 | 5.33E-94 |
| CCL19 | 0.754095 | 1.00E-95 | 8.33E-94 |
| CD74 | 0.754012 | 1.08E-95 | 8.94E-94 |
| PTPRO | 0.753375 | 1.91E-95 | 1.58E-93 |
| STAT5A | 0.752902 | 2.91E-95 | 2.40E-93 |
| CD27 | 0.752643 | 3.67E-95 | 3.00E-93 |
| LRRC33 | 0.752462 | 4.31E-95 | 3.52E-93 |
| INPP5D | 0.752069 | 6.11E-95 | 4.97E-93 |
| STAC3 | 0.752038 | 6.29E-95 | 5.09E-93 |
| PDCD1LG2 | 0.751884 | 7.21E-95 | 5.81E-93 |
| IL2RB | 0.751653 | 8.85E-95 | 7.11E-93 |
| FGD3 | 0.751405 | 1.10E-94 | 8.82E-93 |
| CD72 | 0.749686 | 5.03E-94 | 4.01E-92 |
| CD300C | 0.747874 | 2.46E-93 | 1.95E-91 |
| SIGLEC5 | 0.746845 | 6.02E-93 | 4.75E-91 |
| SIRPB2 | 0.746619 | 7.33E-93 | 5.77E-91 |
| LAT2 | 0.746017 | 1.23E-92 | 9.67E-91 |
| ZEB2 | 0.74532 | 2.25E-92 | 1.76E-90 |
| FCRL3 | 0.745023 | 2.91E-92 | 2.26E-90 |
| SLA2 | 0.744288 | 5.46E-92 | 4.23E-90 |
| LY9 | 0.744279 | 5.51E-92 | 4.25E-90 |
| LTB | 0.743929 | 7.43E-92 | 5.71E-90 |
| MS4A7 | 0.743279 | 1.30E-91 | 9.92E-90 |
| CD79B | 0.74226 | 3.08E-91 | 2.35E-89 |
| SCML4 | 0.742045 | 3.70E-91 | 2.81E-89 |
| GYPC | 0.741886 | 4.23E-91 | 3.21E-89 |
| GNGT2 | 0.741826 | 4.46E-91 | 3.36E-89 |
| NLRC4 | 0.741505 | 5.85E-91 | 4.39E-89 |
| SLC31A2 | 0.741199 | 7.58E-91 | 5.67E-89 |
| P2RX7 | 0.740748 | 1.11E-90 | 8.27E-89 |
| GIMAP8 | 0.739949 | 2.17E-90 | 1.61E-88 |
| TLR10 | 0.739692 | 2.69E-90 | 1.99E-88 |
| GNG2 | 0.739573 | 2.98E-90 | 2.20E-88 |
| NLRP3 | 0.739488 | 3.20E-90 | 2.35E-88 |
| IFFO1 | 0.739108 | 4.40E-90 | 3.22E-88 |
| LRMP | 0.737721 | 1.40E-89 | 1.02E-87 |
| ITGAM | 0.737267 | 2.04E-89 | 1.48E-87 |
| P2RY8 | 0.736261 | 4.69E-89 | 3.40E-87 |
| RASGRP2 | 0.735456 | 9.11E-89 | 6.58E-87 |
| CCR1 | 0.735073 | 1.25E-88 | 8.98E-87 |
| STAP1 | 0.734885 | 1.46E-88 | 1.04E-86 |
| FOXP3 | 0.734818 | 1.54E-88 | 1.10E-86 |
| DOCK10 | 0.732935 | 7.17E-88 | 5.10E-86 |
| RUNX3 | 0.732839 | 7.76E-88 | 5.50E-86 |
| FCGR2C | 0.732577 | 9.60E-88 | 6.78E-86 |
| CASP1 | 0.732549 | 9.82E-88 | 6.91E-86 |
| PPP1R16B | 0.731058 | 3.28E-87 | 2.30E-85 |
| SLAMF7 | 0.730979 | 3.50E-87 | 2.45E-85 |
| CD69 | 0.730926 | 3.65E-87 | 2.54E-85 |
| DNAJC5B | 0.730839 | 3.92E-87 | 2.72E-85 |
| IFI30 | 0.730716 | 4.33E-87 | 2.99E-85 |
| SPIB | 0.729971 | 7.88E-87 | 5.43E-85 |
| RTN1 | 0.729282 | 1.37E-86 | 9.40E-85 |
| EBI3 | 0.729034 | 1.67E-86 | 1.14E-84 |
| PREX1 | 0.729018 | 1.69E-86 | 1.15E-84 |
| CXCR3 | 0.728826 | 1.97E-86 | 1.34E-84 |
| CCR4 | 0.728209 | 3.22E-86 | 2.18E-84 |
| SIRPA | 0.728119 | 3.46E-86 | 2.34E-84 |
| CCR6 | 0.728012 | 3.77E-86 | 2.54E-84 |
| PLA2G2D | 0.727595 | 5.26E-86 | 3.53E-84 |
| ITGA4 | 0.726547 | 1.21E-85 | 8.06E-84 |
| PTGER4 | 0.726049 | 1.79E-85 | 1.19E-83 |
| APOBEC3G | 0.724536 | 5.87E-85 | 3.90E-83 |
| ATP8B4 | 0.724366 | 6.71E-85 | 4.44E-83 |
| CTSS | 0.724273 | 7.22E-85 | 4.76E-83 |
| LILRB3 | 0.723982 | 9.06E-85 | 5.96E-83 |
| P2RY12 | 0.723579 | 1.24E-84 | 8.14E-83 |
| FLT3 | 0.723485 | 1.34E-84 | 8.73E-83 |
| ICAM3 | 0.722772 | 2.33E-84 | 1.52E-82 |
| IL7R | 0.721187 | 7.95E-84 | 5.16E-82 |
| C10orf54 | 0.720909 | 9.86E-84 | 6.38E-82 |
| HLA-DQA1 | 0.720749 | 1.12E-83 | 7.19E-82 |
| FCGR3A | 0.720296 | 1.58E-83 | 1.02E-81 |
| EOMES | 0.720219 | 1.68E-83 | 1.07E-81 |
| CD22 | 0.719387 | 3.18E-83 | 2.03E-81 |
| ANKRD58 | 0.719015 | 4.22E-83 | 2.69E-81 |
| RASSF2 | 0.718561 | 5.97E-83 | 3.79E-81 |
| CXCR5 | 0.717817 | 1.05E-82 | 6.66E-81 |
| CD14 | 0.717305 | 1.55E-82 | 9.80E-81 |
| HK3 | 0.716062 | 3.99E-82 | 2.51E-80 |
| STK10 | 0.714326 | 1.47E-81 | 9.22E-80 |
| ALOX5 | 0.713029 | 3.88E-81 | 2.42E-79 |
| CTLA4 | 0.712712 | 4.91E-81 | 3.06E-79 |
| IRF4 | 0.712706 | 4.93E-81 | 3.06E-79 |
| CD300A | 0.712645 | 5.17E-81 | 3.20E-79 |
| PIK3CD | 0.712379 | 6.29E-81 | 3.88E-79 |
| SLC37A2 | 0.711951 | 8.65E-81 | 5.32E-79 |
| SIGLEC1 | 0.710853 | 1.95E-80 | 1.20E-78 |
| LILRA4 | 0.710344 | 2.84E-80 | 1.73E-78 |
| PRAM1 | 0.710308 | 2.91E-80 | 1.78E-78 |
| GLIPR2 | 0.710223 | 3.10E-80 | 1.89E-78 |
| OSCAR | 0.709505 | 5.26E-80 | 3.19E-78 |
| BCL2A1 | 0.70724 | 2.75E-79 | 1.66E-77 |
| FLT3LG | 0.707229 | 2.78E-79 | 1.67E-77 |
| RAB33A | 0.706783 | 3.84E-79 | 2.30E-77 |
| NAPSB | 0.706744 | 3.95E-79 | 2.36E-77 |
| MATK | 0.706441 | 4.92E-79 | 2.94E-77 |
| VSIG4 | 0.706422 | 4.99E-79 | 2.97E-77 |
| FCGR2B | 0.706297 | 5.46E-79 | 3.24E-77 |
| SLFN12L | 0.706221 | 5.77E-79 | 3.41E-77 |
| CD68 | 0.703962 | 2.94E-78 | 1.73E-76 |
| SAMHD1 | 0.703733 | 3.46E-78 | 2.04E-76 |
| SH2B3 | 0.703628 | 3.73E-78 | 2.19E-76 |
| GBP5 | 0.702441 | 8.73E-78 | 5.10E-76 |
| SUSD3 | 0.70241 | 8.92E-78 | 5.20E-76 |
| CCL5 | 0.702071 | 1.14E-77 | 6.60E-76 |
| CEACAM21 | 0.70047 | 3.53E-77 | 2.05E-75 |
| HSD11B1 | 0.700102 | 4.58E-77 | 2.65E-75 |
| RASGRP4 | 0.699775 | 5.77E-77 | 3.33E-75 |
| GFI1 | 0.699552 | 6.75E-77 | 3.88E-75 |
| MCOLN2 | 0.698847 | 1.11E-76 | 6.35E-75 |
| AP1S2 | 0.698299 | 1.63E-76 | 9.30E-75 |
| GPR55 | 0.697131 | 3.68E-76 | 2.10E-74 |
| HLA-E | 0.696643 | 5.17E-76 | 2.94E-74 |
| MAFB | 0.69651 | 5.68E-76 | 3.22E-74 |
| CD8A | 0.696104 | 7.52E-76 | 4.25E-74 |
| GPR34 | 0.695601 | 1.07E-75 | 6.01E-74 |
| KLRK1 | 0.695475 | 1.16E-75 | 6.54E-74 |
| CCR8 | 0.695293 | 1.32E-75 | 7.39E-74 |
| LYZ | 0.694964 | 1.66E-75 | 9.25E-74 |
| CD163 | 0.694287 | 2.65E-75 | 1.47E-73 |
| VAV1 | 0.693888 | 3.48E-75 | 1.93E-73 |
| LILRA1 | 0.69349 | 4.58E-75 | 2.53E-73 |
| NCF2 | 0.692957 | 6.60E-75 | 3.64E-73 |
| PLCG2 | 0.692104 | 1.18E-74 | 6.51E-73 |
| FOLR2 | 0.691621 | 1.64E-74 | 9.03E-73 |
| SLC9A9 | 0.691488 | 1.80E-74 | 9.85E-73 |
| ALOX5AP | 0.691325 | 2.01E-74 | 1.10E-72 |
| AKNA | 0.691313 | 2.03E-74 | 1.10E-72 |
| IL10 | 0.691224 | 2.15E-74 | 1.17E-72 |
| FMNL3 | 0.690883 | 2.72E-74 | 1.47E-72 |
| NPL | 0.690696 | 3.08E-74 | 1.67E-72 |
| LOC606724 | 0.690567 | 3.37E-74 | 1.81E-72 |
| GPR183 | 0.690319 | 3.98E-74 | 2.14E-72 |
| FASLG | 0.689374 | 7.55E-74 | 4.05E-72 |
| ZBP1 | 0.689238 | 8.28E-74 | 4.42E-72 |
| TRIM22 | 0.688346 | 1.51E-73 | 8.04E-72 |
| MS4A1 | 0.687836 | 2.13E-73 | 1.13E-71 |
| LYL1 | 0.68774 | 2.27E-73 | 1.20E-71 |
| LAX1 | 0.687181 | 3.30E-73 | 1.74E-71 |
| S1PR4 | 0.686436 | 5.43E-73 | 2.86E-71 |
| SLC1A3 | 0.686334 | 5.81E-73 | 3.06E-71 |
| GLIPR1 | 0.686165 | 6.50E-73 | 3.41E-71 |
| HLA-DRB1 | 0.685669 | 9.05E-73 | 4.74E-71 |
| FCRL1 | 0.685359 | 1.11E-72 | 5.80E-71 |
| GAS7 | 0.684968 | 1.44E-72 | 7.51E-71 |
| CNR2 | 0.684498 | 1.97E-72 | 1.02E-70 |
| GBP4 | 0.682206 | 8.92E-72 | 4.62E-70 |
| C5orf56 | 0.681419 | 1.49E-71 | 7.71E-70 |
| RGL4 | 0.681391 | 1.52E-71 | 7.84E-70 |
| C11orf21 | 0.681141 | 1.79E-71 | 9.20E-70 |
| LILRA6 | 0.681034 | 1.92E-71 | 9.84E-70 |
| GPR82 | 0.680392 | 2.92E-71 | 1.49E-69 |
| FAM26F | 0.679814 | 4.25E-71 | 2.17E-69 |
| PDCD1 | 0.679799 | 4.29E-71 | 2.18E-69 |
| BTN3A3 | 0.679671 | 4.66E-71 | 2.37E-69 |
| NKG7 | 0.679113 | 6.70E-71 | 3.39E-69 |
| CCL4 | 0.678686 | 8.83E-71 | 4.46E-69 |
| TRAF1 | 0.67804 | 1.34E-70 | 6.75E-69 |
| RASSF4 | 0.677748 | 1.62E-70 | 8.13E-69 |
| PTGDS | 0.67753 | 1.86E-70 | 9.33E-69 |
| JAK2 | 0.675869 | 5.41E-70 | 2.70E-68 |
| CARD16 | 0.674272 | 1.50E-69 | 7.46E-68 |
| HLA-DOB | 0.673916 | 1.88E-69 | 9.34E-68 |
| MSR1 | 0.672503 | 4.59E-69 | 2.28E-67 |
| GZMA | 0.672409 | 4.88E-69 | 2.41E-67 |
| TBX21 | 0.672379 | 4.97E-69 | 2.45E-67 |
| IL2RA | 0.672291 | 5.25E-69 | 2.59E-67 |
| C1orf200 | 0.672066 | 6.05E-69 | 2.97E-67 |
| PTAFR | 0.671411 | 9.14E-69 | 4.48E-67 |
| TMC8 | 0.670488 | 1.63E-68 | 7.97E-67 |
| BLK | 0.670268 | 1.87E-68 | 9.13E-67 |
| TREM2 | 0.669491 | 3.04E-68 | 1.48E-66 |
| ANKRD44 | 0.669286 | 3.46E-68 | 1.68E-66 |
| ACP5 | 0.669246 | 3.55E-68 | 1.72E-66 |
| CECR1 | 0.669031 | 4.05E-68 | 1.96E-66 |
| LRRC8C | 0.668984 | 4.17E-68 | 2.01E-66 |
| JAK3 | 0.668722 | 4.91E-68 | 2.36E-66 |
| TMEM150B | 0.66835 | 6.19E-68 | 2.97E-66 |
| PTGDR | 0.667196 | 1.27E-67 | 6.05E-66 |
| CLEC17A | 0.666556 | 1.88E-67 | 8.96E-66 |
| C19orf38 | 0.666439 | 2.02E-67 | 9.61E-66 |
| RASGRP3 | 0.66641 | 2.06E-67 | 9.76E-66 |
| POU2F2 | 0.664605 | 6.23E-67 | 2.95E-65 |
| IL18BP | 0.664312 | 7.45E-67 | 3.52E-65 |
| SERPING1 | 0.664086 | 8.56E-67 | 4.03E-65 |
| B2M | 0.663075 | 1.59E-66 | 7.46E-65 |
| PTCRA | 0.663056 | 1.60E-66 | 7.52E-65 |
| TNFRSF8 | 0.662793 | 1.88E-66 | 8.81E-65 |
| HTRA4 | 0.662407 | 2.38E-66 | 1.11E-64 |
| SIRPB1 | 0.662257 | 2.61E-66 | 1.21E-64 |
| CST7 | 0.661248 | 4.80E-66 | 2.23E-64 |
| COTL1 | 0.660874 | 6.02E-66 | 2.79E-64 |
| ADAP2 | 0.659608 | 1.29E-65 | 5.97E-64 |
| HLA-DMA | 0.658566 | 2.41E-65 | 1.11E-63 |
| P2RX1 | 0.658129 | 3.13E-65 | 1.44E-63 |
| RHOH | 0.655771 | 1.27E-64 | 5.83E-63 |
| GPR84 | 0.655451 | 1.53E-64 | 7.03E-63 |
| HLA-B | 0.655424 | 1.56E-64 | 7.13E-63 |
| MPP1 | 0.654653 | 2.46E-64 | 1.12E-62 |
| FCN1 | 0.654606 | 2.53E-64 | 1.15E-62 |
| CXCR4 | 0.65453 | 2.64E-64 | 1.20E-62 |
| C9orf139 | 0.654301 | 3.02E-64 | 1.37E-62 |
| KLRG1 | 0.653271 | 5.54E-64 | 2.50E-62 |
| SNAI3 | 0.652347 | 9.51E-64 | 4.29E-62 |
| PVRIG | 0.651616 | 1.46E-63 | 6.56E-62 |
| CD19 | 0.651417 | 1.63E-63 | 7.34E-62 |
| BCL11B | 0.650534 | 2.73E-63 | 1.22E-61 |
| PPM1M | 0.650284 | 3.16E-63 | 1.41E-61 |
| FCGR2A | 0.650227 | 3.26E-63 | 1.46E-61 |
| CLEC7A | 0.649594 | 4.71E-63 | 2.10E-61 |
| CD40 | 0.648881 | 7.10E-63 | 3.15E-61 |
| C1orf38 | 0.648373 | 9.52E-63 | 4.22E-61 |
| ABI3BP | 0.648148 | 1.08E-62 | 4.79E-61 |
| TMEM140 | 0.648122 | 1.10E-62 | 4.85E-61 |
| CAMK4 | 0.648055 | 1.14E-62 | 5.03E-61 |
| IL18RAP | 0.646691 | 2.50E-62 | 1.10E-60 |
| PLXNC1 | 0.646644 | 2.57E-62 | 1.12E-60 |
| SIGLEC8 | 0.646582 | 2.66E-62 | 1.16E-60 |
| CASS4 | 0.646301 | 3.12E-62 | 1.36E-60 |
| IL4I1 | 0.646183 | 3.34E-62 | 1.45E-60 |
| C17orf60 | 0.646014 | 3.68E-62 | 1.60E-60 |
| CLEC12A | 0.645466 | 5.02E-62 | 2.18E-60 |
| RBP5 | 0.643481 | 1.55E-61 | 6.70E-60 |
| PDE6G | 0.642615 | 2.52E-61 | 1.09E-59 |
| ABCA6 | 0.642059 | 3.45E-61 | 1.49E-59 |
| ZBTB32 | 0.641649 | 4.34E-61 | 1.87E-59 |
| ADORA3 | 0.641126 | 5.82E-61 | 2.50E-59 |
| LILRB5 | 0.6409 | 6.61E-61 | 2.83E-59 |
| ZC3H12D | 0.640557 | 8.01E-61 | 3.42E-59 |
| DOCK11 | 0.640258 | 9.46E-61 | 4.03E-59 |
| LAT | 0.639704 | 1.29E-60 | 5.48E-59 |
| TCL1A | 0.639537 | 1.41E-60 | 6.00E-59 |
| FAM159A | 0.639437 | 1.49E-60 | 6.33E-59 |
| CD101 | 0.6387 | 2.25E-60 | 9.51E-59 |
| PRF1 | 0.637706 | 3.90E-60 | 1.65E-58 |
| TNFRSF9 | 0.637492 | 4.40E-60 | 1.85E-58 |
| MRC1 | 0.63738 | 4.68E-60 | 1.96E-58 |
| BTN3A1 | 0.636197 | 8.97E-60 | 3.76E-58 |
| SIGLEC11 | 0.635935 | 1.04E-59 | 4.33E-58 |
| CCRL2 | 0.635745 | 1.15E-59 | 4.80E-58 |
| CYLD | 0.635624 | 1.23E-59 | 5.12E-58 |
| KCNK13 | 0.634828 | 1.90E-59 | 7.90E-58 |
| SLC2A9 | 0.634815 | 1.91E-59 | 7.94E-58 |
| FPR1 | 0.634809 | 1.92E-59 | 7.95E-58 |
| PLA2G7 | 0.63402 | 2.96E-59 | 1.22E-57 |
| MEI1 | 0.633868 | 3.21E-59 | 1.32E-57 |
| TNFRSF13B | 0.632755 | 5.88E-59 | 2.42E-57 |
| PLA2G4C | 0.632659 | 6.20E-59 | 2.54E-57 |
| PTPN6 | 0.632511 | 6.72E-59 | 2.75E-57 |
| FCRLA | 0.632281 | 7.61E-59 | 3.11E-57 |
| EMR1 | 0.63189 | 9.40E-59 | 3.83E-57 |
| CXCR2P1 | 0.631811 | 9.82E-59 | 4.00E-57 |
| FMNL1 | 0.631733 | 1.02E-58 | 4.16E-57 |
| DENND1C | 0.631092 | 1.45E-58 | 5.87E-57 |
| TNFAIP8 | 0.631035 | 1.49E-58 | 6.04E-57 |
| PARP15 | 0.630918 | 1.59E-58 | 6.42E-57 |
| TMEM229B | 0.630297 | 2.22E-58 | 8.95E-57 |
| PLEKHO1 | 0.630271 | 2.25E-58 | 9.06E-57 |
| C14orf139 | 0.630216 | 2.32E-58 | 9.31E-57 |
| SAMD9L | 0.629722 | 3.02E-58 | 1.21E-56 |
| CD83 | 0.629604 | 3.22E-58 | 1.29E-56 |
| CARD8 | 0.62958 | 3.26E-58 | 1.30E-56 |
| ADORA2A | 0.629563 | 3.29E-58 | 1.31E-56 |
| CFP | 0.629171 | 4.07E-58 | 1.62E-56 |
| APOC2 | 0.628546 | 5.68E-58 | 2.25E-56 |
| PTGS1 | 0.627404 | 1.04E-57 | 4.14E-56 |
| STAT4 | 0.6272 | 1.16E-57 | 4.60E-56 |
| FCER2 | 0.626781 | 1.45E-57 | 5.74E-56 |
| GPNMB | 0.626436 | 1.75E-57 | 6.87E-56 |
| CPVL | 0.625914 | 2.30E-57 | 9.05E-56 |
| SPATC1 | 0.625882 | 2.34E-57 | 9.19E-56 |
| C8orf80 | 0.62518 | 3.40E-57 | 1.33E-55 |
| NAALADL1 | 0.625124 | 3.50E-57 | 1.37E-55 |
| TTC24 | 0.624904 | 3.93E-57 | 1.53E-55 |
| TREML1 | 0.624817 | 4.11E-57 | 1.60E-55 |
| GPSM3 | 0.624591 | 4.63E-57 | 1.80E-55 |
| IRF1 | 0.624345 | 5.28E-57 | 2.04E-55 |
| TLR1 | 0.624316 | 5.36E-57 | 2.07E-55 |
| P2RY14 | 0.624289 | 5.44E-57 | 2.10E-55 |
| GPR141 | 0.624139 | 5.88E-57 | 2.27E-55 |
| CYSLTR2 | 0.62393 | 6.56E-57 | 2.52E-55 |
| C1orf54 | 0.623674 | 7.51E-57 | 2.88E-55 |
| CSF2RA | 0.623654 | 7.59E-57 | 2.90E-55 |
| GM2A | 0.62338 | 8.76E-57 | 3.35E-55 |
| FNBP1 | 0.62306 | 1.04E-56 | 3.95E-55 |
| NOD2 | 0.621975 | 1.83E-56 | 6.96E-55 |
| EMP3 | 0.621804 | 2.00E-56 | 7.60E-55 |
| ENTPD1 | 0.621776 | 2.03E-56 | 7.69E-55 |
| CALHM2 | 0.621761 | 2.04E-56 | 7.74E-55 |
| APOC1 | 0.621612 | 2.21E-56 | 8.34E-55 |
| ARID5A | 0.621308 | 2.59E-56 | 9.76E-55 |
| GZMH | 0.6213 | 2.60E-56 | 9.78E-55 |
| FCRL6 | 0.619804 | 5.65E-56 | 2.12E-54 |
| ATP6V1B2 | 0.619405 | 6.95E-56 | 2.61E-54 |
| LGALS2 | 0.618948 | 8.80E-56 | 3.29E-54 |
| IL3RA | 0.61734 | 2.01E-55 | 7.52E-54 |
| LIPA | 0.616359 | 3.33E-55 | 1.24E-53 |
| ACSM5 | 0.615553 | 5.02E-55 | 1.87E-53 |
| TSPAN32 | 0.61486 | 7.15E-55 | 2.66E-53 |
| CD79A | 0.614619 | 8.08E-55 | 3.00E-53 |
| C1R | 0.614332 | 9.35E-55 | 3.46E-53 |
| CXCL9 | 0.614321 | 9.41E-55 | 3.47E-53 |
| ZNF804A | 0.613846 | 1.20E-54 | 4.41E-53 |
| GPR132 | 0.613637 | 1.33E-54 | 4.90E-53 |
| OLR1 | 0.612366 | 2.53E-54 | 9.29E-53 |
| BTN2A2 | 0.612071 | 2.93E-54 | 1.08E-52 |
| APOB48R | 0.6104 | 6.79E-54 | 2.49E-52 |
| PLD4 | 0.61036 | 6.93E-54 | 2.53E-52 |
| TM6SF1 | 0.610058 | 8.06E-54 | 2.94E-52 |
| RHOG | 0.609638 | 9.94E-54 | 3.62E-52 |
| TM7SF4 | 0.609526 | 1.05E-53 | 3.82E-52 |
| HLA-DQA2 | 0.609459 | 1.09E-53 | 3.94E-52 |
| WDFY4 | 0.609401 | 1.12E-53 | 4.05E-52 |
| HLA-F | 0.609368 | 1.14E-53 | 4.11E-52 |
| RAB8B | 0.60933 | 1.16E-53 | 4.18E-52 |
| ETS1 | 0.609317 | 1.17E-53 | 4.20E-52 |
| GBP1 | 0.608884 | 1.45E-53 | 5.20E-52 |
| SLC8A1 | 0.608846 | 1.48E-53 | 5.29E-52 |
| GMIP | 0.608371 | 1.87E-53 | 6.69E-52 |
| ZNF80 | 0.608071 | 2.17E-53 | 7.76E-52 |
| MCART6 | 0.607192 | 3.35E-53 | 1.20E-51 |
| GAL3ST4 | 0.607046 | 3.61E-53 | 1.28E-51 |
| EMILIN2 | 0.606895 | 3.88E-53 | 1.38E-51 |
| PLXDC2 | 0.6061 | 5.75E-53 | 2.04E-51 |
| C7orf58 | 0.605979 | 6.11E-53 | 2.16E-51 |
| GNAI2 | 0.605695 | 7.02E-53 | 2.48E-51 |
| STAB1 | 0.604949 | 1.01E-52 | 3.58E-51 |
| IL9R | 0.604828 | 1.08E-52 | 3.79E-51 |
| DAB2 | 0.604715 | 1.14E-52 | 4.00E-51 |
| NLRC5 | 0.604481 | 1.28E-52 | 4.48E-51 |
| C12orf59 | 0.603511 | 2.05E-52 | 7.19E-51 |
| CARD9 | 0.603255 | 2.32E-52 | 8.13E-51 |
| TMEM149 | 0.601265 | 6.12E-52 | 2.14E-50 |
| SRGN | 0.601193 | 6.33E-52 | 2.21E-50 |
| BHLHE41 | 0.600926 | 7.21E-52 | 2.51E-50 |
| APOBEC3H | 0.600689 | 8.08E-52 | 2.81E-50 |
| C19orf35 | 0.600687 | 8.09E-52 | 2.81E-50 |
| FCRL4 | 0.600618 | 8.37E-52 | 2.90E-50 |
| CRYBB1 | 0.600288 | 9.81E-52 | 3.39E-50 |
| CCL3 | 0.600204 | 1.02E-51 | 3.53E-50 |

Supplementary Table 2 The drug that correlated with SASH3 expression examine by GDSC database

| symbol | drug | cor | fdr | entrez |
| --- | --- | --- | --- | --- |
| SASH3 | I-BET-762 | -0.54456 | 1.09E-70 | 54440 |
| SASH3 | PIK-93 | -0.53192 | 6.75E-67 | 54440 |
| SASH3 | TPCA-1 | -0.49989 | 7.36E-58 | 54440 |
| SASH3 | PHA-793887 | -0.49316 | 2.96E-56 | 54440 |
| SASH3 | NPK76-II-72-1 | -0.48925 | 3.33E-55 | 54440 |
| SASH3 | Methotrexate | -0.47263 | 4.84E-47 | 54440 |
| SASH3 | NG-25 | -0.47248 | 9.76E-51 | 54440 |
| SASH3 | KIN001-102 | -0.4723 | 7.28E-51 | 54440 |
| SASH3 | BX-912 | -0.45109 | 7.03E-46 | 54440 |
| SASH3 | CAL-101 | -0.4465 | 2.62E-44 | 54440 |
| SASH3 | XMD13-2 | -0.44451 | 2.83E-44 | 54440 |
| SASH3 | AT-7519 | -0.44425 | 3.72E-44 | 54440 |
| SASH3 | Tubastatin A | -0.44397 | 4.91E-44 | 54440 |
| SASH3 | TG101348 | -0.44371 | 3.27E-44 | 54440 |
| SASH3 | JW-7-24-1 | -0.44291 | 4.89E-44 | 54440 |
| SASH3 | CP466722 | -0.44177 | 9.37E-44 | 54440 |
| SASH3 | BIX02189 | -0.44116 | 3.02E-43 | 54440 |
| SASH3 | CAY10603 | -0.43986 | 5.13E-43 | 54440 |
| SASH3 | TL-1-85 | -0.43933 | 4.48E-43 | 54440 |
| SASH3 | BHG712 | -0.43565 | 3.36E-42 | 54440 |
| SASH3 | ZSTK474 | -0.43296 | 1.36E-41 | 54440 |
| SASH3 | CUDC-101 | -0.43052 | 2.87E-40 | 54440 |
| SASH3 | AR-42 | -0.42927 | 1.4E-40 | 54440 |
| SASH3 | SNX-2112 | -0.42435 | 1.8E-39 | 54440 |
| SASH3 | QL-XI-92 | -0.41812 | 1.29E-38 | 54440 |
| SASH3 | GSK1070916 | -0.41385 | 1.05E-36 | 54440 |
| SASH3 | WZ3105 | -0.41352 | 7.99E-38 | 54440 |
| SASH3 | TAK-715 | -0.41251 | 2.39E-37 | 54440 |
| SASH3 | GSK690693 | -0.41186 | 4.31E-37 | 54440 |
| SASH3 | THZ-2-102-1 | -0.40881 | 4.6E-36 | 54440 |
| SASH3 | KIN001-236 | -0.40648 | 5.63E-36 | 54440 |
| SASH3 | BMS345541 | -0.40524 | 4.22E-36 | 54440 |
| SASH3 | PI-103 | -0.4015 | 4.91E-35 | 54440 |
| SASH3 | KIN001-260 | -0.39912 | 9.18E-35 | 54440 |
| SASH3 | QL-X-138 | -0.39788 | 4E-34 | 54440 |
| SASH3 | THZ-2-49 | -0.3906 | 1.07E-32 | 54440 |
| SASH3 | Vorinostat | -0.38561 | 4.18E-30 | 54440 |
| SASH3 | Belinostat | -0.38402 | 5.69E-31 | 54440 |
| SASH3 | OSI-027 | -0.37487 | 2.91E-30 | 54440 |
| SASH3 | PAC-1 | -0.36774 | 5.84E-26 | 54440 |
| SASH3 | Navitoclax | -0.36773 | 3.75E-27 | 54440 |
| SASH3 | KIN001-244 | -0.36761 | 6.7E-29 | 54440 |
| SASH3 | YM201636 | -0.36199 | 5.57E-28 | 54440 |
| SASH3 | Genentech Cpd 10 | -0.35793 | 1.96E-27 | 54440 |
| SASH3 | GSK2126458 | -0.35664 | 5.8E-27 | 54440 |
| SASH3 | Ispinesib Mesylate | -0.35593 | 5.08E-27 | 54440 |
| SASH3 | CX-5461 | -0.34734 | 1.59E-25 | 54440 |
| SASH3 | Y-39983 | -0.34434 | 5.61E-25 | 54440 |
| SASH3 | UNC0638 | -0.34374 | 8.85E-27 | 54440 |
| SASH3 | AZD8055 | -0.33662 | 1.48E-21 | 54440 |
| SASH3 | IPA-3 | -0.33534 | 8.27E-22 | 54440 |
| SASH3 | Phenformin | -0.3349 | 2.73E-23 | 54440 |
| SASH3 | 5-Fluorouracil | -0.33089 | 4.67E-23 | 54440 |
| SASH3 | QL-XII-61 | -0.32681 | 1.58E-10 | 54440 |
| SASH3 | FK866 | -0.3175 | 4.07E-21 | 54440 |
| SASH3 | OSI-930 | -0.3154 | 1.61E-20 | 54440 |
| SASH3 | Masitinib | -0.30287 | 4.32E-19 | 54440 |
| SASH3 | EKB-569 | -0.29962 | 4.02E-18 | 54440 |
| SASH3 | TL-2-105 | -0.29855 | 1.48E-18 | 54440 |

Supplementary Table 3 The drug that correlated with SASH3 expression examine by CTRP database

| symbol | drug | cor | fdr | entrez |
| --- | --- | --- | --- | --- |
| SASH3 | BRD-K30748066 | -0.5026 | 0.001467 | 54440 |
| SASH3 | GSK-J4 | -0.43039 | 0.002245 | 54440 |
| SASH3 | PF-3758309 | -0.39501 | 5.41E-14 | 54440 |
| SASH3 | dinaciclib | -0.39015 | 1.28E-13 | 54440 |
| SASH3 | tozasertib | -0.38786 | 0.00943 | 54440 |
| SASH3 | belinostat | -0.38337 | 1.59E-13 | 54440 |
| SASH3 | SR-II-138A | -0.38173 | 1.55E-27 | 54440 |
| SASH3 | alvocidib | -0.37862 | 2.32E-12 | 54440 |
| SASH3 | CR-1-31B | -0.37834 | 1.52E-26 | 54440 |
| SASH3 | ciclopirox | -0.36591 | 9.1E-25 | 54440 |
| SASH3 | LRRK2-IN-1 | -0.36585 | 1.88E-17 | 54440 |
| SASH3 | I-BET151 | -0.36104 | 4.78E-24 | 54440 |
| SASH3 | narciclasine | -0.35697 | 1.42E-22 | 54440 |
| SASH3 | decitabine | -0.35373 | 4.91E-23 | 54440 |
| SASH3 | SNX-2112 | -0.35263 | 7.21E-23 | 54440 |
| SASH3 | AT13387 | -0.34768 | 3.05E-10 | 54440 |
| SASH3 | Merck60 | -0.34654 | 7.2E-22 | 54440 |
| SASH3 | necrosulfonamide | -0.34606 | 5.48E-15 | 54440 |
| SASH3 | GSK525762A | -0.34441 | 1.54E-21 | 54440 |
| SASH3 | JQ-1 | -0.34344 | 1.5E-21 | 54440 |
| SASH3 | cytarabine hydrochloride | -0.34327 | 1.02E-21 | 54440 |
| SASH3 | BRD-K01737880 | -0.34285 | 0.045649 | 54440 |
| SASH3 | elocalcitol | -0.3418 | 4.72E-20 | 54440 |
| SASH3 | LY-2183240 | -0.33716 | 7.29E-21 | 54440 |
| SASH3 | apicidin | -0.3365 | 6.5E-21 | 54440 |
| SASH3 | GSK461364 | -0.33312 | 4.85E-20 | 54440 |
| SASH3 | omacetaxine mepesuccinate | -0.3328 | 1.21E-14 | 54440 |
| SASH3 | triazolothiadiazine | -0.33211 | 9.75E-21 | 54440 |
| SASH3 | piperlongumine | -0.33178 | 3.45E-20 | 54440 |
| SASH3 | parbendazole | -0.33163 | 1.23E-20 | 54440 |
| SASH3 | PX-12 | -0.33036 | 1.23E-19 | 54440 |
| SASH3 | tivantinib | -0.32861 | 7.61E-10 | 54440 |
| SASH3 | clofarabine | -0.32808 | 4.72E-20 | 54440 |
| SASH3 | teniposide | -0.32739 | 1.36E-10 | 54440 |
| SASH3 | BRD-K66453893 | -0.32368 | 5.87E-19 | 54440 |
| SASH3 | panobinostat | -0.32366 | 2.11E-19 | 54440 |
| SASH3 | vorinostat | -0.32277 | 9.27E-19 | 54440 |
| SASH3 | manumycin A | -0.32231 | 8.1E-19 | 54440 |
| SASH3 | isoliquiritigenin | -0.32172 | 5.54E-05 | 54440 |
| SASH3 | PHA-793887 | -0.32077 | 6.09E-19 | 54440 |
| SASH3 | curcumin | -0.32049 | 2.47E-18 | 54440 |
| SASH3 | BRD-A94377914 | -0.3198 | 8.08E-10 | 54440 |
| SASH3 | COL-3 | -0.3187 | 2.13E-13 | 54440 |
| SASH3 | vincristine | -0.31723 | 2.54E-19 | 54440 |
| SASH3 | KPT185 | -0.31512 | 1.5E-13 | 54440 |
| SASH3 | PL-DI | -0.31511 | 8.35E-18 | 54440 |
| SASH3 | CHM-1 | -0.31427 | 4.09E-18 | 54440 |
| SASH3 | NSC95397 | -0.31352 | 7.11E-16 | 54440 |
| SASH3 | docetaxel | -0.3116 | 2.18E-08 | 54440 |
| SASH3 | doxorubicin | -0.31049 | 3.58E-18 | 54440 |
| SASH3 | sotrastaurin | -0.31026 | 2.05E-12 | 54440 |
| SASH3 | ISOX | -0.30881 | 3.03E-17 | 54440 |
| SASH3 | BRD-K61166597 | -0.30762 | 6.31E-16 | 54440 |
| SASH3 | isoevodiamine | -0.30512 | 4.82E-17 | 54440 |
| SASH3 | BI-2536 | -0.30488 | 1.37E-17 | 54440 |
| SASH3 | KX2-391 | -0.30461 | 9.18E-17 | 54440 |
| SASH3 | etoposide | -0.30358 | 6.18E-17 | 54440 |
| SASH3 | SB-743921 | -0.30161 | 3.53E-17 | 54440 |
| SASH3 | leptomycin B | -0.30041 | 4.37E-17 | 54440 |
| SASH3 | Compound 23 citrate | -0.30027 | 1.15E-15 | 54440 |
| SASH3 | tacedinaline | -0.30009 | 8.25E-12 | 54440 |
| SASH3 | PRIMA-1 | -0.29988 | 1.6E-15 | 54440 |
| SASH3 | MK-1775 | -0.29961 | 6.92E-16 | 54440 |
| SASH3 | ouabain | -0.29842 | 3.03E-16 | 54440 |
| SASH3 | entinostat | -0.29795 | 6.95E-16 | 54440 |
| SASH3 | rigosertib | -0.29633 | 1.72E-15 | 54440 |
| SASH3 | GW-405833 | -0.29606 | 1.01E-15 | 54440 |
| SASH3 | gemcitabine | -0.29534 | 1.01E-14 | 54440 |
| SASH3 | BRD-K34222889 | -0.29519 | 5.89E-16 | 54440 |
| SASH3 | dacarbazine | -0.29446 | 3.35E-15 | 54440 |
| SASH3 | indisulam | -0.29348 | 6.72E-15 | 54440 |
